# Antibiotic‐Induced Gut Microbiota Dysbiosis Modulates Host Transcriptome and m^6^A Epitranscriptome via Bile Acid Metabolism

**DOI:** 10.1002/advs.202307981

**Published:** 2024-05-07

**Authors:** Meng Yang, Xiaoqi Zheng, Jiajun Fan, Wei Cheng, Tong‐Meng Yan, Yushan Lai, Nianping Zhang, Yi Lu, Jiali Qi, Zhengyi Huo, Zihe Xu, Jia Huang, Yuting Jiao, Biaodi Liu, Rui Pang, Xiang Zhong, Shi Huang, Guan‐Zheng Luo, Gina Lee, Christian Jobin, A. Murat Eren, Eugene B Chang, Hong Wei, Tao Pan, Xiaoyun Wang

**Affiliations:** ^1^ School of Life Sciences South China Normal University Guangzhou 510631 China; ^2^ Guangzhou Institutes of Biomedicine and Health Chinese Academy of Sciences Guangzhou 510530 China; ^3^ College of Animal Science and Technology Huazhong Agricultural University Wuhan 430070 China; ^4^ State Key Laboratory of Quality Research in Chinese Medicine Macau University of Science and Technology Taipa Macau 999078 China; ^5^ MOE Key Laboratory of Gene Function and Regulation State Key Laboratory of Biocontrol School of Life Sciences Sun Yat‐sen University Guangzhou 510275 China; ^6^ Guangdong Provincial Key Laboratory of Microbial Safety and Health State Key Laboratory of Applied Microbiology Southern China Institute of Microbiology Guangdong Academy of Sciences Guangzhou 510070 China; ^7^ College of Animal Science and Technology Nanjing Agricultural University Nanjing 210095 China; ^8^ Faculty of Dentistry The University of Hong Kong Hong Kong SAR China; ^9^ Department of Microbiology and Molecular Genetics Chao Family Comprehensive Cancer Center University of California Irvine School of Medicine Irvine CA 92697 USA; ^10^ Department of Medicine University of Florida College of Medicine Gainesville FL 32610 USA; ^11^ Helmholtz Institute for Functional Marine Biodiversity 26129 Oldenburg Germany; ^12^ Institute for Chemistry and Biology of the Marine Environment University of Oldenburg 26129 Oldenburg Germany; ^13^ Department of Medicine Knapp Center for Biomedical Discovery The University of Chicago Knapp Center for Biomedical Discovery Chicago IL 60637 USA; ^14^ Department of Biochemistry and Molecular Biology The University of Chicago Chicago IL 60637 USA; ^15^ University of Chinese Academy of Sciences Beijing 100049 China

**Keywords:** bile acids, epitranscriptome, gut microbiota, N6‐Methyladenosine, transcriptome

## Abstract

Gut microbiota can influence host gene expression and physiology through metabolites. Besides, the presence or absence of gut microbiome can reprogram host transcriptome and epitranscriptome as represented by *N* ^6^‐methyladenosine (m^6^A), the most abundant mammalian mRNA modification. However, which and how gut microbiota‐derived metabolites reprogram host transcriptome and m^6^A epitranscriptome remain poorly understood. Here, investigation is conducted into how gut microbiota‐derived metabolites impact host transcriptome and m^6^A epitranscriptome using multiple mouse models and multi‐omics approaches. Various antibiotics‐induced dysbiotic mice are established, followed by fecal microbiota transplantation (FMT) into germ‐free mice, and the results show that bile acid metabolism is significantly altered along with the abundance change in bile acid‐producing microbiota. Unbalanced gut microbiota and bile acids drastically change the host transcriptome and the m^6^A epitranscriptome in multiple tissues. Mechanistically, the expression of m^6^A writer proteins is regulated in animals treated with antibiotics and in cultured cells treated with bile acids, indicating a direct link between bile acid metabolism and m^6^A biology. Collectively, these results demonstrate that antibiotic‐induced gut dysbiosis regulates the landscape of host transcriptome and m^6^A epitranscriptome via bile acid metabolism pathway. This work provides novel insights into the interplay between microbial metabolites and host gene expression.

## Graphical abstract

Yang et al. report that gut dysbiosis induced by antibiotics can lead to tissue‐specific reprogramming of host transcriptome and epitranscriptome via alterations in bile acid metabolism. Mechanistically, it is shown that m^6^A writers are regulated in animals treated with antibiotics and in cultured cells treated with bile acids, indicating a direct link between bile acid metabolism and m^6^A biology.

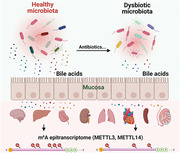

## 1 Introduction

The gut microbiota plays an important role in maintaining host physiology, including digestion and nutrient uptake, metabolism, development, and immunity.^[^ ^1^, ^2^, ^3^, ^4^ ^]^ At the molecular level, gut microbiota contributes to host physiology through the production of a myriad of metabolites (e.g., neurotransmitters, vitamins, bile acids, etc.), which exert their effects within the host as signaling molecules and substrates in metabolic reactions.^[^ ^5^ ^]^ Microbial metabolites are absorbed across the host gut and can interact with any cell of our body through systemic circulation.^[^ ^6^ ^]^ As such, disruption of the gut microbiota network, a process often refers to dysbiosis is associated with many diseases such as inflammatory bowel diseases, neurological diseases, and metabolic disorders.^[^ ^7^, ^8^ ^]^

The alteration of gut microbiome in relationship to host gene expression can occur in multiple ways. At the transcriptome level, epigenetic changes enable the host cells to adapt their transcriptional program to environmental cues.^[^ ^9^ ^]^ Recently, gut microbiota‐mediated epigenetic regulation has been shown as one type of host‐microbe interactions. The links between epigenetic changes and gut microbiota can be mediated by microbiota‐derived metabolites that act as substrates and cofactors for key epigenetic enzymes in the host.^[^ ^10^ ^]^ However, molecular mechanisms by which the gut microbiota chemically regulate the host gene expression and physiology remain largely unknown.^[^ ^11^, ^12^, ^13^, ^14^ ^]^

*N* ^6^‐methyladenosine (m^6^A) is the most abundant mammalian mRNA modification that affects all aspects of mRNA life including stability, splicing, translation, and decay.^[^ ^15^, ^16^ ^]^ The m^6^A modification is installed by writer proteins in mammalian cells in response to environmental cues and thus could readily respond to the type and the state of gut microbiota. We previously showed that mouse gut microbiota reprograms the host mRNA methylome and tRNA methylome in multiple tissues.^[^ ^17^, ^18^ ^]^ This host‐microbe interaction was also observed by other groups, suggesting that the RNA epitranscriptomic regulation represents an additional level of host‐microbe interactions.^[^ ^19^, ^20^, ^21^ ^]^ Although the role of gut microbiota in regulating host RNA epitranscriptome has been recognized, however the specific microbiota‐derived metabolites mediating the epitranscriptomic response remain to be elucidated.

Here, we investigated the impact of altered gut microbiota and associated metabolites on host transcriptome and m^6^A epitranscriptome in animals and cells. Our metabolomic results demonstrate that antibiotic‐induced dysbiosis in the gut microbiota significantly changes the bile acid metabolism in conventional mice, which was verified using fecal microbiota transplantation (FMT) in germ‐free (GF) mice. We further showed that specific bile acids treatment directly altered the expression of m^6^A machinery proteins in mammalian cells. Our results demonstrate a key role of the bile acid metabolism in regulating host transcriptome and m^6^A epitranscriptome with dysbiotic gut microbiota.

## 2 Results

### 2.1 Characterization of Gut Dysbiosis Mouse Models Induced by Antibiotics

**Figure 1:**
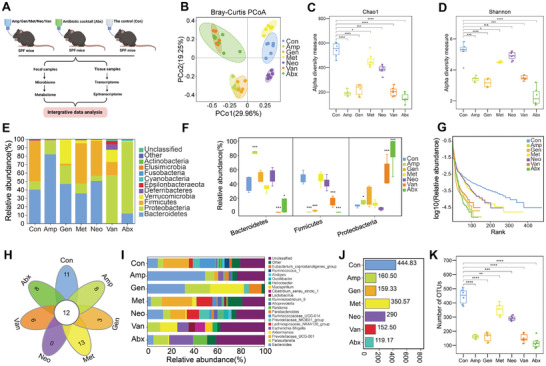
Gut dysbiosis mouse models induced by antibiotics and the microbial composition analysis. A) Schematic diagram of gut dysbiosis models and multi‐omics analysis in this study. Antibiotic‐treated groups include single antibiotic including ampicillin (Amp), gentamicin (Gen), metronidazole (Met), neomycin (Neo) and vancomycin (Van), or an antibiotic cocktail (Abx). Mice treated with distilled water were included as the control (Con). B) Principal coordinate analysis (beta‐diversity) of 16S rRNA gene sequencing datasets showing microbial compositions in different antibiotic‐treated groups (*n* = 6–7 each). C) Chao1 analysis index showing alpha‐diversity of microbial compositions in different antibiotic‐treated groups (*n* = 6–7 each). D) Shannon analysis index showing alpha‐diversity of microbial compositions in different antibiotic‐treated groups (*n* = 6–7 each). E) Phylum‐level taxonomic profiles of fecal microbiotas in different antibiotic‐treated groups (*n* = 6–7 each). F) Relative abundance of three dominant phyla in different antibiotic‐treated groups (*n* = 6–7 each). G) Rank graphs showing relative abundance of microbial compositions in different antibiotic‐treated groups (*n* = 6–7 each). H) Venn diagrams showing microbial compositions at genus level in different antibiotic‐treated groups (*n* = 6–7 each). I) Relative abundance of microbial compositions at genus level in different antibiotic‐treated groups (*n* = 6–7 each). J) Number of operational taxonomic unit (OTU) in different antibiotic‐treated groups (*n* = 6–7 each). K) Boxplots shows the difference in the number of OTUs between each of antibiotic‐treated groups and the control group (*n* = 6–7 each). *P < 0.05, **P < 0.01, ***P < 0.001, ****P < 0.0001, Student's *t* test.

To identify specific bacteriome regulating host transcriptome and epitranscriptome, we constructed several gut dysbiosis mouse models (**Figure** **1**A). We exposed conventional SPF mice to different single antibiotic dose including ampicillin (Amp), gentamicin (Gen), metronidazole (Met), neomycin (Neo) and vancomycin (Van), or to an antibiotic cocktail (Abx, mixture of the above five antibiotics) via drinking water for 40 days. Mice treated with only distilled water were included as the control (Con). On day 40, we collected fecal and tissue samples for multi‐omics profiling including microbiome, metabolome, transcriptome, m^6^A epitranscriptome, and proteome.

We first analyzed the composition of gut commensals in fecal samples from different antibiotic exposure by 16S ribosomal RNA (rRNA) amplicon sequencing. Principal coordinate analysis (PCoA) showed that the beta‐diversity in antibiotic treatment groups (Amp, Gen, Met, Neo, Van, Abx) had substantial divergence compared to the no antibiotic control, with biological replicates well clustered within each group (Figure 1B). The diversity indices (Sob, Chao1, Shannon, and Simpson) revealed that the control group maintained excellent diversity and richness, while the diversity and richness in other groups diminished, some to a great extent (Figure 1C,D; Figure S1A–C, Supporting Information). As expected, relative abundance analysis at the phylum level indicated that the dominant phyla in fecal samples of control mice were *Bacteroidetes*, *Firmicutes*, and *Proteobacteria* (Figure 1E). Compared to the control, all antibiotics greatly disturbed the composition of the gut microbiota, especially the three dominant phyla (Figure 1E–G). Our data revealed that *Firmicutes*/*Bacteroidetes* ratios were significantly perturbed by nearly all antibiotic treatments (Figure S1D, Supporting Information), and the relative abundance of *Proteobacteria* in antibiotic treatment groups was increased compared to the control (Figure 1E,F; Figure S1A, Supporting Information). At the genus level, the microbial compositions in antibiotic treatment groups differed greatly (Figure 1H,I; Figure S1E,F, Supporting Information), and the number of operational taxonomic units (OTUs) was also significantly reduced by antibiotic treatments (Figure 1J,K). We also performed microbial composition analysis for Abx group and Con group, and the results showed that microbial compositions in Abx group were obviously reduced by antibiotic cocktail treatment (Figure S2, Supporting Information). However, after 40 days of treated many bacteria still could be detected in the fecal samples, especially the Gram‐negative bacteria and pathogenic bacteria. Overall, our microbial analysis suggested that antibiotic treatments significantly reduced the bacterial diversity of gut microbiota.

The dominant gut microbial phyla for mammals are *Firmicutes*, *Bacteroides*, *Proteobacteria*, *Actinobacteria*, and *Verrucomicrobia*,^[^ ^22^ ^]^ among which *Firmicutes* and *Bacteroidetes* are the two major phyla. The *Firmicutes*/*Bacteroidetes* ratio has been widely used as an indicator of microbial homeostasis for human and mouse gut microbiota, and changes in *Firmicutes*/*Bacteroidetes* ratio have been proposed as indicators for gut dysbiosis.^[^ ^23^, ^24^ ^]^ In addition, *Proteobacteria* is considered a microbial signature of gut dysbiosis.^[^ ^25^ ^]^ The *Firmicutes*/*Bacteroidetes* ratio was altered by all antibiotic treatments, bactericidal activity also varied among different antibiotics. For example, ampicillin and gentamicin had strong bactericidal capacity against *Firmicutes*, while gentamicin also increased the relative abundance of *Verrucomicrobia* (Figure 1E; Figure S1A, Supporting Information). Collectively, we conclude that our mouse models of gut dysbiosis were constructed successfully and each antibiotic generated different gut microbiota dysbiosis.

### 2.2 Antibiotic‐Induced Gut Microbiota Disturbance Causes Imbalance of Bile Acid Metabolism

Previous studies suggested that microbial metabolites can affect host transcriptome and epitranscriptome by acting as substrates and cofactors for various key enzymes.^[^ ^5^, ^11^, ^26^ ^]^ We performed untargeted metabolome measurements to examine whether gut microbiota disturbance by antibiotics changed microbial‐derived metabolites in fecal samples. Since the microbial compositions in ampicillin‐induced and ampicillin‐containing cocktail groups had the least diversity after antibiotic exposure, we focused our metabolome study on mice feces from Con, Amp, and Abx groups.

**Figure 2:**
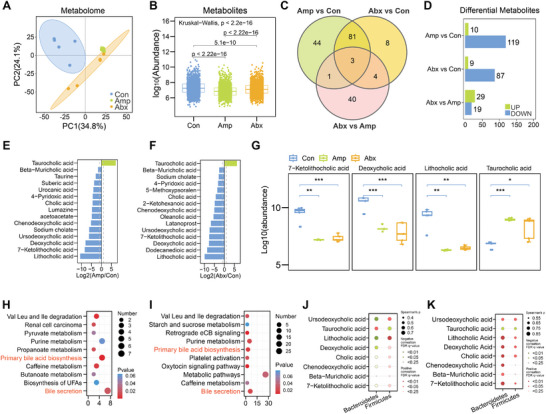
Antibiotic‐induced gut dysbiosis causes imbalance of bile acid metabolism. A) PCA plot of fecal metabolome datasets in three groups (Con, Amp, Abx. *n* = 5 for each group). B) Abundance of metabolites identified from fecal samples in three groups (Con, Amp, Abx. *n* = 5 each). Kruskal‐Wallis Test was used for statistical analysis. C) Venn diagram depicting the shared or unique number of antibiotic‐treatment associated metabolites in three between‐group comparisons (*n* = 5 each). D) The number of antibiotic‐enriched and depleted metabolites related to Amp and Abx (*n* = 5 each). E) Top 15 differential metabolites with ‐log2(Amp/Con) > 3 (*n* = 5 each). Down‐ and up‐regulated metabolites were in blue and green, respectively. F) Top 15 differential metabolites with ‐log2(Abx/Con) > 3 (*n* = 5 each). Down‐ and up‐regulated metabolites were in blue and green, respectively. G) The top 3 down‐regulated bile acids and top 1 up‐regulated bile acid in Amp and Abx groups compared to the Con group (*n* = 5 each). H) Functional pathways involved in the differential metabolites between Amp and Con group (*n* = 5 each), bile‐acid‐related pathways were highlighted in red. I) Functional pathways of differential metabolites between Abx and Con group (*n* = 5 each), bile acids pathways were highlighted in red. J) Correlation analysis of differential metabolites with *Firmicutes* and *Bacteroidetes* between Amp group and Con group (*n* = 5 each). K) Correlation analysis of differential metabolites with *Firmicutes* and *Bacteroidetes* between Abx group and Con group (*n* = 5 each). *P < 0.05, **P < 0.01, ***P < 0.001, Student's *t* test.

We identified a total of 1725 metabolites by untargeted metabolome analysis (Table S1, Supporting Information). PCA results showed that the three experimental groups (Con, Amp, and Abx) were well separated, suggesting that microbe‐derived metabolites under the Amp or Abx treatment had significant differences from those in the Con group (**Figure** **2**A). Moreover, the overall abundance of metabolites in Amp or Abx group was significantly lower than that in the Con group (Figure 2B). We analyzed differential metabolites between every pair of treatment groups and identified 129, 96 and 48 differential metabolites for Amp/Con, Abx/Con, and Abx/Amp, respectively (Figure 2C). Most of the differential metabolites were downregulated upon Amp or Abx treatment (Figure 2D). Strikingly, we found that bile acids were the most obviously changed metabolites upon antibiotic‐treatment compared to the control (Figure 2E,F). Among them, the levels of lithocholic acid, deoxycholic acid and 7‐ketolithocholic acid were severely decreased by Amp or Abx treatment, while taurocholic acid was significantly increased by Amp or Abx treatment (Figure 2G). The Kyoto Encyclopedia of Genes and Genomes (KEGG) pathway analysis of differential metabolites revealed that pathways such as bile secretion and primary bile acid biosynthesis were enriched for both Amp/Con (Figure 2H) and Abx/Con comparison (Figure 2I). Next, we performed correlation analysis between the altered metabolites and the pronouncedly changed gut microbiota at phylum level and genus level. The decreased metabolites mediated by Amp (Figure 2J; Figure S1G, Supporting Information) or Abx (Figure 2K; Figure S1H, Supporting Information) closely correlated with the abundance of *Firmicutes* and *Bacteroidetes*, suggesting that the down‐regulated metabolites might be due to changes of *Firmicutes*/*Bacteroidetes*.

### 2.3 FMT Verifies the Association of Antibiotic‐Induced Gut Dysbiosis with Bile Acid Metabolism

**Figure 3:**
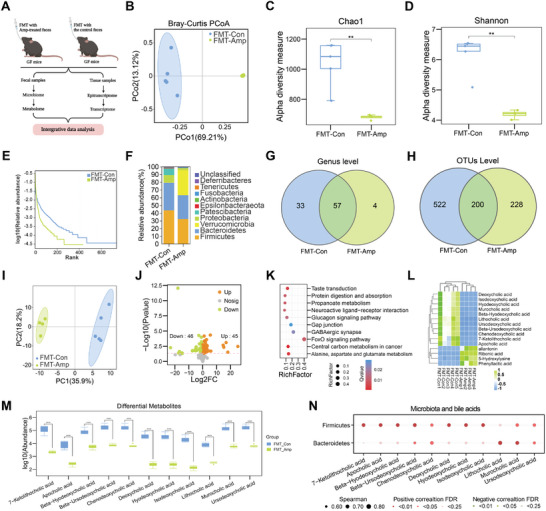
Microbiome and metabolome profiling after fecal microbial transplantation experiments in GF mice. A) Schematic diagram of FMT experiments and multi‐omics analysis. GF mice were colonized with fecal samples from ampicillin‐treated mice (FMT‐Amp) or the control mice (FMT‐Con), followed by multi‐omics profiling and integrative data analysis. B) Principal coordinate analysis (beta‐diversity) of 16S rRNA gene sequencing datasets showing between‐sample difference in microbial compositions in FMT‐Amp group and FMT‐Con group (*n* = 4–5 for each group). C) Chao1 analysis showing alpha‐diversity of microbial compositions in FMT‐Amp group and FMT‐Con group (*n* = 4–5 each). D) Shannon analysis showing alpha‐diversity of microbial compositions in FMT‐Amp group and FMT‐Con group (*n* = 4–5 each). E) Rank graphs showing relative abundance of microbial compositions in FMT‐Amp group and FMT‐Con group (*n* = 4–5 each). F) Relative abundance of microbial compositions at phylum level in FMT‐Amp group and FMT‐Con group (*n* = 4–5 each). G) Venn diagrams showing microbial compositions at genus level in FMT‐Amp group and FMT‐Con group (*n* = 4–5 each). H) Venn diagrams showing numbers of OTUs in FMT‐Amp group and FMT‐Con group. I) PCA results of fecal metabolome datasets in FMT‐Amp group and FMT‐Con group (*n* = 4–5 each). J) Volcano plot showing up‐ and down‐regulated metabolites in FMT‐Amp group and FMT‐Con group (*n* = 4–5 each). K) Functional pathways of differential metabolites in FMT‐Amp group and FMT‐Con group (*n* = 4–5 each). L) Top 15 differential metabolites in FMT‐Amp group and FMT‐Con group (*n* = 4–5 each). M) Box plot shows the differential bile acids in FMT‐Amp group and FMT‐Con group (*n* = 4–5 each). N) Correlation analysis of differential bile acids with *Firmicutes* and *Bacteroidetes* between FMT‐Amp group and FMT‐Con group (*n* = 4–5 each). *P < 0.05, **P < 0.01, ***P < 0.001, ****P < 0.0001, Student's *t* test.

To determine whether bile acid changes were responsible for Amp‐induced microbiota dysbiosis, we performed FMT by colonizing fecal samples from the Con group or Amp group into GF mice followed by microbiome and metabolome analysis (**Figure** **3**A). PCoA analysis of 16S rRNA gene sequencing data showed a clear microbiota separation between FMT‐Con group and FMT‐Amp group (Figure 3B). The diversity of gut microbiota in FMT‐Amp group was significantly lower than FMT‐Con group (Figure 3C–E; Figure S3A–D, Supporting Information). Similar with the microbial structures in conventional SPF mice, *Firmicutes* and *Bacteroidetes* were still the two dominant phyla in fecal samples of GF mice for FMT‐Con group and FMT‐Amp group (Figure 3F). We also analyzed the microbial structures at the genus level (Figure 3G; Figure S3E–G, Supporting Information) and OTU level (Figure 3H), both of which showed reduced richness of gut microbiota in the FMT‐Amp mice compared to the FMT‐Con mice. In addition to phylum level and genus level, we also analyzed microbial structures at other levels (class, family, order, species) for antibiotic‐treated mice (Figure S4A–D, Supporting Information) and FMT mice (Figure S4E–H, Supporting Information). We also performed functional analysis on the gut microbiome for both antibiotic‐treated mice (Figure S4I, Supporting Information) and FMT mice (Figure S4J, Supporting Information), most enriched functions are overlapped between Amp group and FMT‐Amp, compared to the group. Overall, microbial profiling between FMT recipient and antibiotic‐treated mice showed similar gut microbiota dysbiosis, where *Firmicutes* were consistently depleted in both antibiotic treatment experiments (Figure S3H, Supporting Information) and FMT experiments (Figure S3I, Supporting Information).

Next, we performed targeted metabolome study focusing on bile acids. Fecal metabolomes from two groups were different as shown by PCA analysis (Figure 3I). Among 219 metabolites, we identified a total of 209 and 213 metabolites for FMT‐Amp and FMT‐Con groups, respectively (Table S2, Supporting Information). Among the differential metabolites, 46 and 45 metabolites either decreased or increased in the FMT‐Amp group compared to the FMT‐Con group (Figure 3J, Supporting Information). KEGG pathway analysis of those differential metabolites revealed that diverse metabolic pathways were enriched for FMT‐Amp/Con comparison (Figure 3K). We further analyzed differential bile acids between the two groups and found that most differential bile acids were lower in FMT‐Amp samples compared to FMT‐Con group (Figure 3L,M), and most of the differential bile acids strongly correlated with *Firmicutes* (Figure 3N). Importantly, we found that the three bile acids, lithocholic acid, deoxycholic acid and 7‐ketolithocholic acid, were down‐regulated both in FMT‐Amp and Amp group (Figure S3J, Supporting Information). We also observed that microbiome and metabolome profiles in GF mice were different from that in SPF mice, presumably due to the variable colonization ability of several microbes in GF mice and SPF mice. However, our FMT experiments in GF mice generally recapitulated the microbiome and metabolome profiles observed in antibiotic‐mediated gut microbial dysbiosis in the SPF mice.

### 2.4 The Impact of Gut Microbiota on Mouse Transcriptome after Antibiotic Treatment and FMT

**Figure 4:**
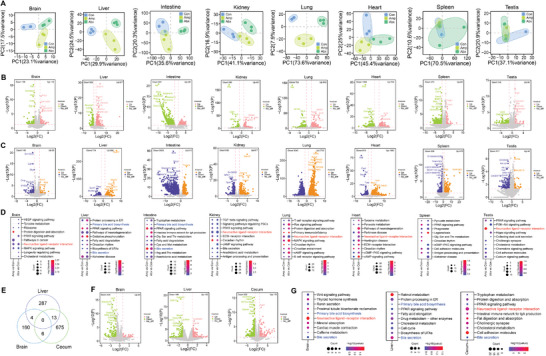
Transcriptome‐wide impact of gut microbiota on mouse tissue‐specific gene expression. A) PCA results of mouse mRNA transcriptome datasets for eight tissues (brain, liver, intestine, kidney, lung, heart, spleen, testis) in three groups (Con, Amp, Abx. *n* = 3 each). B) Volcano plot showing up‐ and down‐regulated genes for eight tissues in ampicillin treatment compared to the control (*n* = 3 each). Genes with expression level of fold change ≥ 2 and *P* < 0.05 are presented. C) Volcano plot showing up‐ and down‐regulated genes for eight tissues in antibiotic cocktail treatment compared to the control (*n* = 3 each). Genes with expression level of fold change ≥ 2 and *P* < 0.05 are presented. D) KEGG analysis showing enriched pathways with differentially expressed genes for eight tissues in three groups (*n* = 3 each). E) Venn diagram showing the differences and overlaps of genes for three tissues between FMT‐Amp and FMT‐Con groups (*n* = 3 each). F) Volcano plot showing up‐ and down‐regulated genes for three tissues between FMT‐Amp and FMT‐Con groups (*n* = 3 each). Genes with expression level of fold change ≥ 2 and *P* < 0.05 are presented. G) KEGG analysis showing enriched pathways with differentially expressed genes for three tissues between FMT‐Amp and FMT‐Con groups (*n* = 3 each). Neuroactive ligand‐receptor interaction pathway and bile secretion pathway were highlighted in red and blue, respectively.

After the establishment of gut dysbiosis models using antibiotics and FMT, we investigated the impact of changed gut microbiota and metabolites on host gene expression. We first analyzed the mRNA transcriptome profiles of eight mouse tissues (Figure S5A, Supporting Information) in the three above‐mentioned groups (Con, Amp and Abx). PCA results of transcriptome datasets (brain, liver, intestine, kidney, lung, heart, spleen, and testis) showed that samples between groups were well separated (**Figure** **4**A), indicating the global impact of gut microbiota dysbiosis on mouse transcriptome. Among all tissues, many differentially expressed genes were up‐ or down‐regulated by ampicillin (Figure 4B; Table S3, Supporting Information) or antibiotic cocktail (Figure 4C; Table S3, Supporting Information) in a tissue‐specific manner. The number of differentially expressed genes varied from hundreds to thousands for different tissues. KEGG analysis suggested that large number of tissue‐specific pathways were affected by gut dysbiosis induced by antibiotics (Figure 4D). Transcriptomic analysis for brain, liver, and cecum tissues from FMT experiments in GF mice also revealed profound difference between FMT‐Amp and FMT‐Con (Figure 4E,F; Table S3, Supporting Information), and various pathways were affected by gut dysbiosis (Figure 4G). Notably, both SPF transcriptome and GF transcriptome showed that bile acid metabolism‐associated pathways were enriched for differentially expressed genes after antibiotic treatment in brain, liver and cecum/intestine tissues. In addition, neuroactive ligand‐receptor interaction pathway was found to be enriched in many tissues after antibiotic treatment.

For the brain tissue, we also collected samples treated with other antibiotics treatments and performed mRNA transcriptome analysis among samples (Con, Gen, Met, Neo, Van). Hundreds of transcripts with differential expression levels were found upon the treatment of different antibiotics in the brain (Figure S5B–G and Table S3, Supporting Information). Gene Ontology and KEGG analysis indicated that differentially expressed genes upon antibiotic treatment were enriched in neural signaling pathways (Figure S5H,I, Supporting Information). We also determined the impact of gut dysbiosis on host gene expression at protein level using quantitative proteomic approach (Figure S5J and Table S4, Supporting Information). Among thousands of detected proteins in ampicillin‐treated and the control groups, we identified 588, 250, and 245 differentially expressed proteins for brain, liver, and intestine, respectively (Figure S5K, Supporting Information). Among those proteins, many were enriched in either neural signaling or metabolism‐associated pathways (Figure S5L, Supporting Information). Collectively, our results from transcriptome and proteome confirmed the impact of antibiotic‐induced gut dysbiosis on mouse gene expression in multiple tissues.

### 2.5 Gut Microbiota Perturbation Reshapes Mouse Brain mRNA m^6^A Epitranscriptome

**Figure 5:**
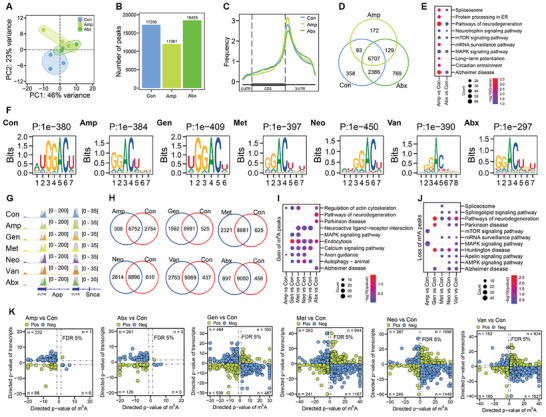
Gut dysbiosis reshapes host brain mRNA m^6^A epitranscriptome and gene expression. A) PCA results of mouse brain mRNA m^6^A epitranscriptome datasets in three groups (Con, Amp, Abx. *n* = 3 each). B) Numbers of peaks identified in three groups (*n* = 3 each). C) The frequency of m^6^A peaks distributed across the mRNA regions (5′UTR, CDS, 3′UTR) in three groups (*n* = 3 each). D) Venn diagram showing numbers of m^6^A peak‐containing genes in three groups (*n* = 3 each). E) KEGG analysis showing functional pathways of differentially abundant m^6^A peak‐containing genes between antibiotic‐treatment groups and the control (*n* = 3 each). F) Representative consensus motifs and corresponding *P* values of m^6^A peaks identified in different groups (*n* = 3 each). G) Integrative genomics viewer (IGV) depicting representative sequencing coverage of m^6^A‐IP (different colors) and Input (gray) showing differential m^6^A peaks on the transcripts *Snca* and *Pink1*. H) The gain and loss peak numbers in different antibiotic treatment groups compared to the control (*n* = 3 for each group). I) KEGG analysis showing functional pathways of genes gaining m^6^A peaks in different groups (*n* = 3 each). J) KEGG analysis showing functional pathways of genes losing m^6^A peaks in different groups (*n* = 3 each). K) Correlation analysis of mouse brain m^6^A epitranscriptome with transcriptome in different groups compared to the control (*n* = 3 each).

To further investigate the impact of gut microbiota and metabolites on host gene expression, we profiled the mRNA m^6^A epitranscriptome in mouse brain tissues, which harbor relatively high m^6^A contents.^[^ ^17^ ^]^ We built m^6^A‐seq (MeRIP‐seq) libraries using the m^6^A‐seq2 protocol, which employs multiplexed m^6^A‐immunoprecipitation of barcoded RNAs and greatly increases the throughput of sequencing samples. The m^6^A‐seq libraries and RNA‐seq libraries using mouse brain mRNAs were sequenced, and the profiles of brain mRNA m^6^A epitranscriptome from three groups (Con, Amp, Abx) were assessed. PCA results of MeRIP‐seq datasets showed that samples between three groups were well separated (**Figure** **5**A), indicating the global impact of gut microbiota dysbiosis on mouse brain mRNA m^6^A epitranscriptome. Overall, 17 256, 11 981, and 18 455 m^6^A peaks were identified in mouse brain tissues for Con, Amp, Abx, respectively (Figure 5B). Consistent with previous studies,^[^ ^15^, ^17^ ^]^ m^6^A peaks in all samples were located in 5′UTR, CDS and 3′UTR, with an enrichment around the stop codon regions (Figure 5C). The m^6^A peaks identified were enriched in 9544, 7101, 9991 genes for Con, Amp, Abx, respectively (Table S5, Supporting Information), with common and specific genes for each group (Figure 5D). KEGG pathway enrichment showed that the gut microbiota imbalance led to enrichments in brain's neural signaling‐related pathways (Figure 5E).

We also profiled the mRNA m^6^A epitranscriptome in mouse brain tissues treated with other antibiotics (Gen, Met, Neo, Van), and the results supported the influence of gut microbiota dysbiosis on mouse brain mRNA m^6^A epitranscriptome (Figure S6A–C, Supporting Information). The well‐established mammalian m^6^A motif RRACH (where R represents G or A; H represents A, C, or U), particularly GGACU, was enriched in the identified peaks in all groups of samples with high confidence (Figure 5F), indicating the validity and reliability of our MeRIP‐seq datasets. *Snca* and *Pink1* are well‐characterized genes related to neurodegenerative diseases (e.g., Alzheimer's disease, Parkinson's disease). Integrative genomics viewer (IGV) tracks revealed that m^6^A peaks in 3′UTR regions of these genes were indeed altered in different antibiotic treatment groups (Figure 5G). We also analyzed the gain and loss of m^6^A peaks upon individual antibiotic treatment condition (Figure 5H; Table S6, Supporting Information). Neural signaling pathways were enriched for genes either gaining or losing m^6^A peaks, and antibiotic‐specific pathways were also present (Figure 5I,J).

To understand the role of gut dysbiosis and altered m^6^A modifications in regulating brain gene expression, we performed correlation analysis of mouse brain m^6^A epitranscriptome with the corresponding transcriptome. The results suggested that hundreds of transcripts were either positively or negatively correlated to m^6^A modification (Figure 5K). We continued to analyze the m^6^A peak densities, most antibiotic treatments affected the m^6^A peak density on exons (Figure S6D, Supporting Information), and the patterns were consistent with m^6^A peak numbers. In addition, m^6^A peak intensity on transcripts also indicated that m^6^A clusters were alterable by antibiotic treatments (Figure S6E, Supporting Information). These findings collectively suggest that antibiotic treatments significantly altered mRNA m^6^A epitranscriptome and transcriptome in the host brain.

### 2.6 Gut Dysbiosis Reprograms Host Liver and Intestine mRNA m^6^A Epitranscriptome

**Figure 6:**
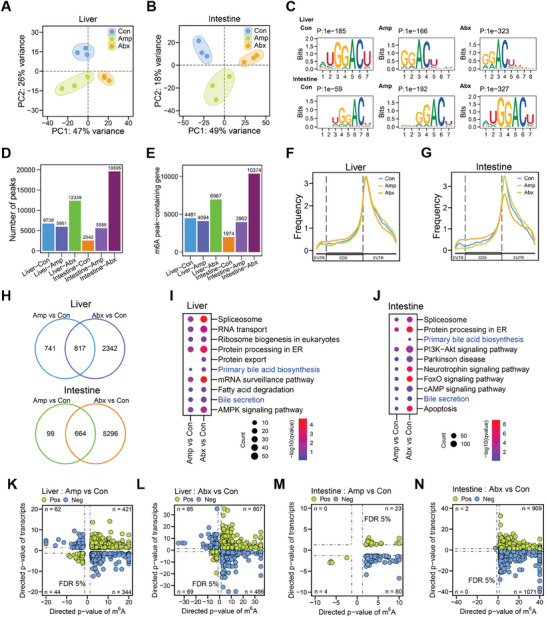
Reprogramming of host liver and intestine mRNA m^6^A epitranscriptome by gut dysbiosis. A) PCA plot of mouse liver mRNA m^6^A epitranscriptomes in three groups (Con, Amp, Abx. *n* = 3 each). B) PCA plot of mouse intestine mRNA m^6^A epitranscriptomes in three groups (*n* = 3 each). C) Representative consensus motifs and corresponding *P* values of m^6^A peaks identified from liver and intestine in three groups (*n* = 3 each). D) Numbers of peaks identified from liver and intestine in three groups (*n* = 3 each). E) Numbers of m^6^A peak‐containing genes in liver and intestine of three groups (*n* = 3 each). F) The frequency of m^6^A peaks distributed across the mRNA regions (5′UTR, CDS, 3′UTR) in liver of three groups (*n* = 3 each). G) The frequency of m^6^A peaks distributed across the mRNA regions (5′UTR, CDS, 3′UTR) in intestine of different groups (*n* = 3 each). H) Venn diagram showing the differences and overlaps of m^6^A peak‐containing genes between different groups (*n* = 3 each). I) KEGG analysis showing functional pathways of different m^6^A peak‐containing genes in liver of three groups (*n* = 3 each), bile acid metabolism pathways were highlighted in blue. J) KEGG analysis showing functional pathways of different m^6^A peak‐containing genes in intestine of three groups (*n* = 3 each), bile acid metabolism pathways were highlighted in blue. K) Correlation analysis of mouse liver m^6^A epitranscriptome with transcriptome in Amp group compared to the control (*n* = 3 each). L) Correlation analysis of mouse liver m^6^A epitranscriptome with transcriptome in Abx group compared to the control (*n* = 3 each). M) Correlation analysis of mouse intestine m^6^A epitranscriptome with transcriptome in Amp group compared to the control (*n* = 3 each). N) Correlation analysis of mouse intestine m^6^A epitranscriptome with transcriptome in Abx group compared to the control (*n* = 3 each).

To expand our understanding of gut dysbiosis on host gene expression, we also conducted MeRIP‐seq and compared the m^6^A methylome of the liver and intestine tissues from Con, Amp, and Abx groups, respectively. PCA results indicated that the three groups were well separated in the liver (**Figure** **6**A) and intestine (Figure 6B). The GGACU motif was also enriched in the identified peaks from the liver and intestine of all three groups with high confidence (Figure 6C). For the liver tissue, 6739, 5951, and 12 339 m^6^A peaks were identified in Con, Amp, and Abx, respectively (Figure 6D). The m^6^A peak number in liver tissue decreased by ampicillin treatment but increased by antibiotic cocktail treatment, which had the same trend as this change in the brain tissue. For the intestine tissue, 2540, 5589, and 19 595 m^6^A peaks were identified in Con, Amp and Abx, respectively (Figure 6E). The m^6^A peak number in intestine tissue significantly increased by both ampicillin and antibiotic cocktail treatment. As expected, the m^6^A modification sites in the liver and intestine were mainly distributed in 5′UTR, CDS, and 3′UTR, with an enrichment around stop codon and 3′UTR (Figure 6F). Notably, m^6^A methylome profile in the intestine appeared to be more dynamic than the liver tissue in terms of peak number and peak distribution patterns upon antibiotic treatments (Figure 6G).

Next, we analyzed the profiles of transcripts with m^6^A modifications in liver and intestine tissues. In total, m^6^A peaks identified in different groups were enriched in 4094–6967 transcripts for the liver and 1974–10374 transcripts for the intestine (Figure 6H; Table S7, Supporting Information). KEGG pathway enrichment of transcripts with m^6^A modifications showed that both ampicillin‐induced and antibiotic cocktail‐induced gut microbiota imbalance led to significant changes in diverse pathways including spliceosome, protein processing in ER and mRNA surveillance (Figure 6I,J). Correlation analysis suggested that large number of transcripts were correlated with m^6^A modification transcriptome‐wide (Figure 6K–N). We also analyzed the gain and loss of m^6^A peaks (Table S6, Supporting Information) for the liver and intestine upon antibiotic treatments (Figure S7A,B, Supporting Information), various key functional pathways were enriched for the two tissues (Figure S7C,D, Supporting Information). In the analysis of m^6^A peak density on exons (Figure S7E, Supporting Information), both ampicillin‐induced and antibiotic cocktail‐induced gut dysbiosis altered exonic m^6^A peak densities in either liver or intestine. The m^6^A peak intensities on transcripts were also variable by antibiotic treatments (Figure S7F, Supporting Information).

In addition to the effect of gut microbiota on host m^6^A epitranscriptome in SPF mice, we also performed m^6^A epitranscriptomic study using brain, liver, and cecum tissues from FMT‐Con and FMT‐Amp in GF mice (Figure S8A–C, Supporting Information). Similar with the results from SPF mice, the m^6^A peak numbers were different between FMT‐Con and FMT‐Amp in three tissues (Figure S8D, Supporting Information), and the GGACU motif was enriched in the identified peaks with high confidence (Figure S8E, Supporting Information). The patterns of host m^6^A epitranscriptome reprogramming in FMT experiments were similar with that in antibiotic treatment experiments. We also analyzed the profiles of transcripts with m^6^A modifications for three tissues from FMT experiments (Figure S8F,G, Supporting Information). The KEGG enrichment analysis revealed that bacterial colonization using Amp‐treated fecal samples affected diverse biological pathways in these three tissues (Figure S8H–J, Supporting Information). IGV tracks on genes *Snca* and *Hsp90ab1* supported that m^6^A peaks on those genes were altered in three tissues (Figure S8K). Correlation analysis of FMT datasets also suggested that many transcripts were correlated with m^6^A modification transcriptome‐wide for three tissues (Figure S8L–N, Supporting Information). Overall, our results from FMT experiments confirmed that gut dysbiosis reprograms m^6^A epitranscriptome in both proximal (liver and cecum) and distal tissue (brain) in a tissue‐specific manner.

### 2.7 Tissue‐Specific Epitranscriptomic −Regulation of m^6^A Writers by Gut Dysbiosis

**Figure 7:**
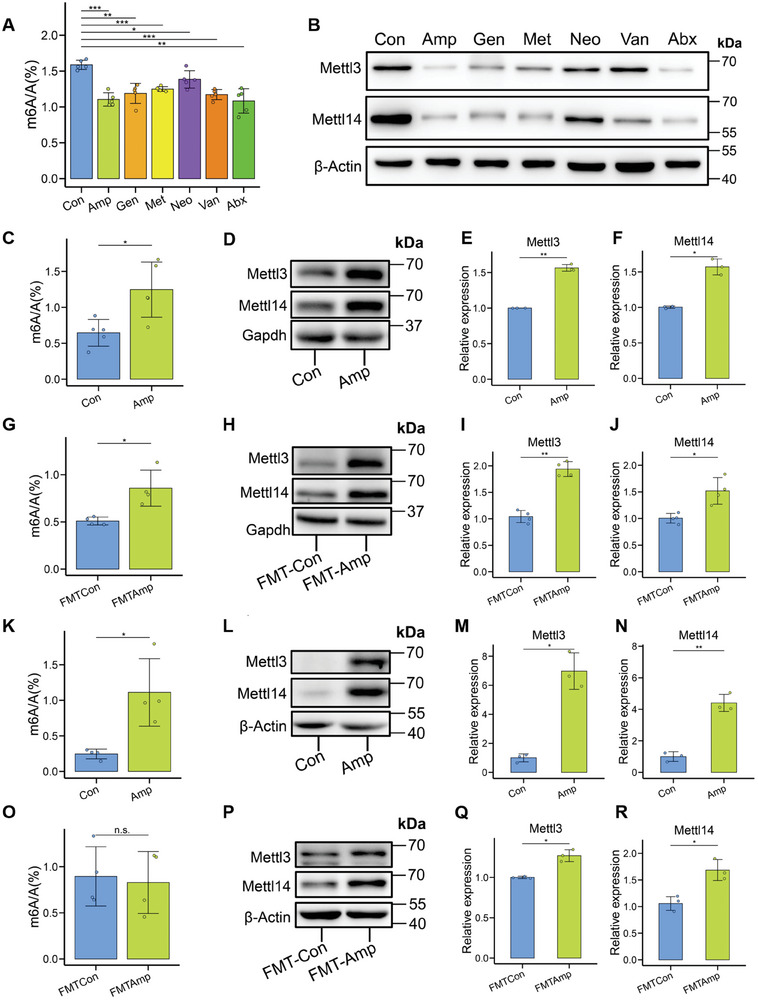
Gut dysbiosis regulates m^6^A modification and m^6^A writers in mouse tissues. A) LC‐MS/MS analysis of m^6^A/A ratios in the brain poly(A)‐selected RNAs from different groups (*n* = 5 each). *P < 0.05, **P < 0.01, ***P < 0.001, Student's *t* test. B) Western blot of mRNA m^6^A writer proteins in brain from different antibiotic treatments (*n* = 3 each). C) LC‐MS/MS analysis of m^6^A A^−1^ ratios in the liver poly(A)‐selected RNAs from Amp group and Con group, respectively (*n* = 5 each). *P < 0.05, Student's *t* test. D) Western blot analysis of mRNA m^6^A writer proteins in liver from Amp group and Con group, respectively (*n* = 3 each). E) Relative expression of METTL3 in liver from Amp group and Con group, respectively (*n* = 3 each). **P < 0.01, Student's *t* test. F) Relative expression of METTL14 in liver from Amp group and Con group, respectively (*n* = 3 each). *P < 0.05, Student's *t* test. G) LC‐MS/MS analysis of m^6^A A^−1^ ratios in the liver poly(A)‐selected RNAs from FMT‐Amp and FMT‐Con, respectively (*n* = 4 each). *P < 0.05, Student's *t* test. H) Western blot analysis of mRNA m^6^A writer proteins in liver from FMT‐Amp and FMT‐Con, respectively (*n* = 4 each). I) Relative expression of METTL3 in liver from FMT‐Amp and FMT‐Con, respectively (*n* = 4 each). **P < 0.01, Student's *t* test. J) Relative expression of METTL14 in liver from FMT‐Amp and FMT‐Con, respectively (*n* = 4 each). *P < 0.05, Student's *t* test. K) LC‐MS/MS analysis of m^6^A/A ratios in the cecum poly(A)‐selected RNAs from Amp group and Con group, respectively (*n* = 4 each). *P < 0.05, Student's *t* test. L) Western blot analysis of mRNA m^6^A writer proteins in cecum from Amp group and Con group, respectively (*n* = 3 each). M) Relative expression of METTL3 in cecum from Amp group and Con group, respectively (*n* = 3 each). *P < 0.05, Student's *t* test. N) Relative expression of METTL14 in cecum from Amp group and Con group, respectively (*n* = 3 each). **P < 0.01, Student's t test. O) LC‐MS/MS analysis of m^6^A A^−1^ ratios in the cecum poly(A)‐selected RNAs from FMT‐Amp and FMT‐Con, respectively (*n* = 4 each). P) Western blot analysis of mRNA m^6^A writer proteins in cecum from FMT‐Amp and FMT‐Con, respectively (*n* = 3 each). Q) Relative expression of METTL3 in cecum from FMT‐Amp and FMT‐Con, respectively (*n* = 3 each). **P < 0.01, Student's *t* test. R) Relative expression of METTL14 in cecum from FMT‐Amp and FMT‐Con, respectively (*n* = 3 each). ***P < 0.001, Student's *t* test.

To obtain mechanistic understanding of m^6^A epitranscriptomic changes induced by gut dysbiosis, we first performed m^6^A analysis using Liquid Chromatography‐tandem Mass Spectrometry (LC‐MS/MS) to determine the m^6^A A^−1^ ratios in poly(A)‐selected RNAs, we observed that the m^6^A A^−1^ ratios in brain tissue were significantly decreased by antibiotic treatments (**Figure** **7**A). Mammalian mRNA m^6^A modification is dynamically regulated by the writer enzyme complex containing METTL3 and METTL14 proteins. We analyzed the levels of the mRNA m^6^A writer proteins in mouse brain exposed to various antibiotics, and we observed that METTL3 and METTL14 were significantly downregulated by antibiotic‐induced gut dysbiosis (Figure 7B; Figure S9A,B, Supporting Information), indicating that gut dysbiosis indeed regulate mouse brain m^6^A writing with different patterns. In addition, we observed that gut dysbiosis induced by most antibiotics tend to downregulate the reader YTHDC1 and the eraser FTO (Figure S9C, Supporting Information), as well as methyl donor‐associated proteins (METTL16, MAT1A, and MAT2A, Figure S9D, Supporting Information).

In our animal experiments, we recorded phenotypes when we collected the tissues from antibiotic treatment experiments or FMT experiments. The phenotypic difference of organ relative weight was visible in the liver and cecum (Figure S9E,F, Supporting Information). In addition to the weight change, we found that the cecum was obviously enlarged in antibiotic‐treated mice compared to the control mice (Figure S9G, Supporting Information). We then analyzed the levels of mRNA m^6^A modification and mRNA m^6^A writer proteins in mouse liver and cecum tissues. For the liver tissue, both m^6^A A^−1^ ratio and m^6^A writer proteins were upregulated by ampicillin treatment (Figure 7C–F) or FMT experiments (Figure 7G–J). For the cecum tissue by ampicillin treatment, m^6^A A^−1^ ratio and m^6^A writer proteins were also upregulated by ampicillin treatment (Figure 7K–N) or FMT experiments (Figure 7O–R). Overall, our results indicate that the m^6^A A^−1^ ratio and m^6^A writer proteins can be regulated directly by antibiotic‐induced gut dysbiosis for liver and cecum. The phenotypes and the responses of m^6^A machinery to different antibiotic treatments tend to be tissue‐specific, which were in line with the tissue‐specific MeRIP‐seq data for m^6^A patterns.

### 2.8 Bile Acids Treatments in Mammalian Cells Alter the Expression of m^6^A Writers

**Figure 8:**
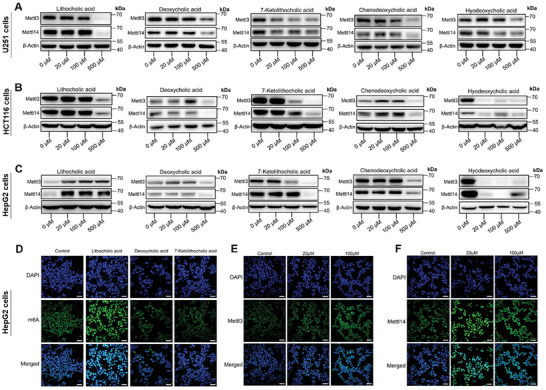
Bile acids treatments in mammalian cells alter the expression of m^6^A writers. A) Western blot analysis of mRNA m^6^A writer proteins in human glioblastoma U251 cells treated with various concentrations of five different bile acids (*n =* 2 or 3 biological replicates). B) Western blot analysis of mRNA m^6^A writer proteins in human colon cancer HCT116 cells treated with various concentrations of five different bile acids (*n =* 2 or 3 biological replicates). C) Western blot analysis of mRNA m^6^A writer proteins in human liver carcinoma HepG2 cells treated with various concentrations of five different bile acids (*n =* 2 or 3 biological replicates). D) Representative images of immunofluorescence analysis showing m^6^A levels in HepG2 cells treated with or without lithocholic acid, deoxycholic acid, and 7‐Ketolithocholic acid at 500 µm. *n* = 3 biological replicates. Scale bars, 40 µm. E) Representative images of immunofluorescence analysis showing the expression of mRNA m^6^A writer METTL3 in HepG2 cells treated with lithocholic acid (0, 20, 100 µm). *n* = 3 biological replicates. Scale bar, 40 µm. F) Representative images of immunofluorescence analysis showing the expression of mRNA m^6^A writer METTL14 in HepG2 cells treated with lithocholic acid (0, 20, and 100 µm). *n* = 3 biological replicates. Scale bar, 40 µm.

Emerging evidence indicates that various metabolites and metabolic pathways are involved in m^6^A mRNA modification.^[^ ^27^, ^28^ ^]^ Like all enzyme reactions, m^6^A writing is dynamically regulated by various substrates and co‐factors. Our metabolomic results from our gut microbiota dysbiosis models showed high level of changes in the microbial metabolites, in particular bile acids. We therefore treated three types of mammalian cells with various bile acids (lithocholic acid, deoxycholic acid, 7‐ketolithocholic acid, chenodeoxycholic acid, and hyodeoxycholic acid) to test whether bile acids directly affect host m^6^A epitranscriptome via the alternation of m^6^A writer proteins. For human glioblastoma U251 cells (**Figure** **8**A; Figure S10A,B, Supporting Information) and human colon cancer HCT116 cells (Figure 8B; Figure S10C,D, Supporting Information), the results strongly supported that the m^6^A writers METTL3 and METTL14 were down‐regulated upon the addition of bile acids, especially at high concentrations.

For human liver carcinoma HepG2 cells with different bile acids, the results showed that the mRNA m^6^A writers METTL3 and METTL14 can be down‐regulated by most bile acids, except lithocholic acid (Figure 8C; Figure S10E,F, Supporting Information). We further analyzed total m^6^A levels in HepG2 cells by immunofluorescence analysis using m^6^A antibody (Figure 8D). Cells were treated with lithocholic acid, deoxycholic acid, and 7‐Ketolithocholic acid, which were identified as the main altered metabolites in our gut dysbiosis mice models. Consistently, total m^6^A levels were down‐regulated by deoxycholic acid and 7‐Ketolithocholic acid, but up‐regulated by lithocholic acid (Figure 8D). The immunofluorescence analysis using METTL3 or METTL14 antibody further confirmed that the treatment of lithocholic acid indeed up‐regulated the expression levels of both METTL3 and METTL14 (Figure 8E,F).

**Figure 9:**
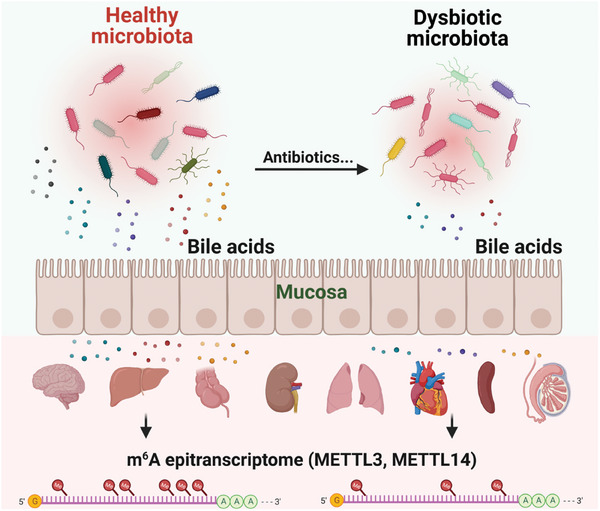
Proposed working model from this study. The status of gut microbiota and microbiota‐derived bile acids can be changed from balance to dysbiosis by environmental factors (e.g., antibiotics). Subsequently, the expression levels of m^6^A writer proteins (METTL3, METTL14, etc.) in host tissues are perturbed, which will reshape transcriptome and m^6^A epitranscriptome. The image is created by BioRender.

Taken together, we propose a working model for this study (**Figure** **9**). Under healthy gut microbiota conditions, microbiota‐derived bile acids are absorbed through the mucosa and circulated to host tissues to maintain mRNA methylation levels and gene expression by regulating expression levels of m^6^A machinery proteins (METTL3, METTL14, etc.). Under dysbiotic microbiota conditions induced by environmental factors (e.g., antibiotics), microbiota‐derived bile acids are reduced to alter the expression levels of m^6^A writer proteins in host tissues, which lead to the reshaping of host transcriptome and m^6^A epitranscriptome.

## 3 Discussion

Recently, our knowledge of the impact of the gut microbiota on the host has greatly increased, especially with the advances in multi‐omics technologies. Given the influence of gut microbiota on host cellular function through metabolites, changes in the composition of the gut microbiota are certain to alter host gene expression and can cause diseases.^[^ ^12^ ^]^ Elucidating the molecular mechanisms by which the gut microbiota interacts with the host physiology is crucial for understanding the role of the microbiota in health and disease.

In this study, we demonstrate that gut dysbiosis in mice induces tissue‐specific reprogramming of host mRNA m^6^A epitranscriptome via bile acid metabolism, which was further verified by fecal microbiota colonization experiment in GF mice. Mechanistically, we demonstrated that the reshaping of mRNA m^6^A epitranscriptome in animal tissues was accompanied by the changes in m^6^A machinery enzymes, which could be reproduced in cultured cells treated with specific bile acids. m^6^A represents the most abundant mammalian mRNA modification that affects diverse biological processes including development, tumorigenesis, circadian clock, and immune response.^[^ ^15^ ^]^ While previous studies support a key role of gut microbiota in regulating host m^6^A epitranscriptome and gene expression,^[^ ^17^, ^19^, ^20^, ^21^, ^29^, ^30^, ^31^ ^]^ the underlying molecular mechanisms for this regulation in the context of host‐microbe interaction are still unclear. Our results here provide a comprehensive landscape of host mRNA m^6^A epitranscriptome in multiple tissues associated with specific metabolites and microbiota.

The diversity and composition of the gut microbiome have been studied for over a decade, but their precise roles in influencing host physiology and maintaining tissue homeostasis are still under intense investigation.^[^ ^32^, ^33^ ^]^ In this study, we found that ampicillin‐induced gut dysbiosis led to unbalanced *Firmicutes*/*Bacteroidetes* ratios and reduced production of secondary bile acids. Bile acids are synthesized from cholesterol in the liver and further metabolized by the gut microbiota into secondary bile acids to exert biological functions.^[^ ^34^ ^]^ It has been well established that the gut microbiota has crucial effects on bile acid metabolism by promoting deconjugation, dehydrogenation, and dehydroxylation of primary bile acids in the distal small intestine and colon, thus regulating the chemical diversity of bile acids.^[^ ^35^, ^36^ ^]^ In our study, we found that lithocholic acid, deoxycholic acid, cholic acid, and 7‐ketolithocholic acid were significantly decreased in Amp‐treated or Abx‐treated mice fecal samples, whereas taurocholic acid was increased in these mice. Our results are consistent with previous studies showing that most of bile acids are lower in GF mice feces or antibiotic‐treated mice feces.^[^ ^37^, ^38^, ^39^ ^]^ Importantly, we have verified our findings in GF mice without antibiotic perturbance and the observed effects in SPF mice were also observed in FMT experiments in GF mice, which ruled out the side effects that might arise from the actions of antibiotics in SPF mice. Previous reports revealed that *Firmicutes* are the main source of bile acid‐producing microbiota, given that *Firmicutes* have large amount of bile salt hydrolase genes comparing to other microbial phyla including *Actinobacteria*,* Bacteroidetes*,* Euryarchaeota*,* and Proteobacteria*, etc.^[^ ^40^ ^]^ Taken together, our microbiome and metabolome results suggest that the bile acid metabolism in antibiotic experiments and FMT experiments was indeed altered with the change in bile acid‐producing microbiota.

Our study indicates that bile acids are the main metabolites regulating host transcriptome and m^6^A epitranscriptome in gut dysbiosis. Bile acids have been shown to influence host DNA and histone methylation during gene transcription.^[^ ^41^, ^42^ ^]^ Recent reports showed that bile acids affect mRNA m^6^A modification by targeting m^6^A catalytic enzymes under disease conditions.^[^ ^43^, ^44^ ^]^ However, the bridge linking bile acid metabolism with host RNA methylome in the context of gut dysbiosis was lacking prior to this study. For the first time, our results added bile acids as the molecular link between gut microbiota and host mRNA m^6^A epitranscriptome. The complete atlas of microbial metabolites and m^6^A methylome reshaped by gut dysbiosis from this study provide an important clue to fill the gap between gut microbiota and the host mRNA epitranscriptome. Our results also have potential therapeutic implications to treat metabolic diseases or other dysbiosis‐related diseases. Maintenance of balanced gut microbiota is essential for intestinal homeostasis and human health, and microbiota‐mediated unbalanced epigenetic or epitranscriptomic signatures are related to many diseases.^[^ ^45^, ^46^, ^47^ ^]^ Thus, our results using the antibiotic‐induced gut dysbiosis support the cautions of antibiotics usage in treating diseases. Also, abnormal bile acids and other metabolites have been suggested as disease biomarkers and targeting these metabolites could present new approaches for treating diseases.^[^ ^48^, ^49^, ^50^ ^]^ Thus, targeting host RNA epitranscriptomic machinery using bile acids or bile acids‐producing microbiota could be an effective and precise approach as a treatment option of bile acid‐related human diseases.

At the molecular level, we demonstrated that the levels of m^6^A machinery proteins and methyl donor‐associated proteins were altered by the gut dysbiosis in animal and bile acid treatments in cultured cells. However, our mechanistic understanding of microbiota‐host RNA epitranscriptomic interactions underlying this regulation is still limited, which will require further investigation. For example, the regulatory patterns of gut microbiota and microbiota‐derived metabolites on host m^6^A methylome and m^6^A machinery proteins tend to be tissue‐specific. Besides, other host factors (age, food, gender, etc.) could also complicate the regulatory process. In addition, our metabolomic analysis reveals numerous non‐bile acid metabolites that were altered in dysbiotic microbiota, which could also impact on host RNA epitranscriptome. In previous study, bile acid was reported to modulate microRNA m^6^A modification by binding to METTL3 and affecting the formation of METTL3‐METTL14‐WTAP complex.^[^ ^43^ ^]^ Whether similar mechanism could be applied to mRNA m^6^A epitranscriptome regulated by bile acids, experiments are required to investigate exact mechanisms in future studies. Future work will be needed to assess the effect of these metabolites on the host RNA epitranscriptome and gene expression. Future work will also include an extensive investigation of whether microbiota‐derived metabolites are involved in the host epitranscriptome other than the m^6^A modification such as pseudouridine.

## 4 Experimental Section

### Ethics Statement

The protocol for animal experiment was reviewed and approved by Institutional Animal Care and Use Committee (IACUC) of South China Normal University (protocol code SCNU‐SLS‐2022‐009).

### Antibiotics Treatment Experiments in Conventional Mice

Specific pathogen free (SPF) mice (male, 6‐weeks old, C57BL/6J) used in this study were kept under a 12‐h light‐dark cycle. SPF mice were purchased from SPF (Beijing) Biotechnology Co., Ltd., and housed in individually ventilated cages for autoclaved food and drinking water. To induce gut dysbiosis models with different microbial compositions, mice were given single antibiotic treatment for 40 days via drinking water including ampicillin (Amp, 1 g L^−1^), gentamicin (Gen, 1 g L^−1^), metronidazole (Met, 1 g L^−1^), neomycin (Neo, 1 g L^−1^), and vancomycin (Van, 0.5 g L^−1^), respectively. Mice were also treated with antibiotic cocktail (Abx): a combination of ampicillin, metronidazole, vancomycin, gentamicin and neomycin with the above concentrations for 40 days. The control mice (Con) were only given drinking water for 40 days. Mice (*n* = 6 each for Con/Amp/Gen/Van/Abx group, and *n* = 7 each for Met/Neo group) were sacrificed to collect tissues and stool samples, samples were immediately dispensed to cryotubes and stored at −80 °C until used.

### Bacterial Colonization in GF Mice

Germ‐free (GF) mice (male, 4‐weeks old, C57BL/6J) were generated and provided by the GF animal platform of Huazhong Agricultural University (Wuhan, China), these mice were maintained in a sterile Trexler‐type isolator, and the mice were housed in a pathogen‐free mouse population (temperature, 25 ± 2 °C; relative humidity, 45–60%; photoperiod, 12 h day^−1^; photoperiod 06:30–18:30), and access to autoclaved food and drinking water. SPF mice and ampicillin‐treated SPF mice were used as donors in the fecal microbial transplantation (FMT) experiments. The fecal microbial suspension was prepared as described in previous study.^[^ ^51^ ^]^ Briefly, fresh feces from SPF mice and ampicillin‐treated SPF mice were homogenized and diluted fivefold in sterile potassium phosphate buffer (0.1 m, pH 7.2) containing 15% glycerol (v/v). Fecal microbial suspension was then immediately dispensed to cryotubes and stored at −80 °C. For FMT experiments, GF mice were randomly allocated into two groups (FMT‐Con and FMT‐Amp, respectively). These mice were inoculated orally with 0.2 mL of fecal microbial suspension, once every day for seven days. Additional 2 mL aliquots were spread on the fur of each mouse. After 7 days of bacterial colonization, mice were reared in a sterile environment until 8‐weeks old and then sacrificed for fecal sample collection (*n* = 4 each for FMT‐Amp group and *n* = 5 each for FMT‐Con group).

### Fecal 16S rRNA Gene Sequencing and Microbial Analysis

Bacterial DNAs were extracted from fresh fecal samples of mice treated with different antibiotics and V4 region of the 16S rRNA gene was amplified for sequencing. Fecal samples of GF mice after FMT experiments were collected and frozen until bacterial DNA was extracted and V3‐V4 region of the 16S rRNA gene were amplified for sequencing. Typically, microbial DNA from 150 to 200 mg of fecal samples was extracted using HiPure Stool DNA Kit (Magen, Guangzhou, China) according to the manufacturer's protocol. High‐throughput sequencing was performed on the Novaseq 6000 platform of Gene Denovo Biotechnology Co. Ltd. in Guangzhou. Quantification was performed on an ABI StepOnePlus real‐time PCR system (Life Technologies, USA) before loading into the sequencer. Purified amplicons were pooled in equimolar for paired‐end sequencing (PE250) on Illumina platform according to standard protocols.

For microbial composition analysis, Illumina paired‐end reads were merged using FLASH (version 1.2.11) software^[^ ^52^ ^]^ with a minimum overlap of 10 bp and a mismatch error rate of 2%, FASTP software^[^ ^53^ ^]^ was used to obtain high‐quality reads for analysis. High‐quality reads were aligned to operational taxonomic units (OTUs) with 97% similarity using the UPARSE (version 9.2.64) software,^[^ ^54^ ^]^ and Silva (bacteria) database (version 132)^[^ ^55^ ^]^ was used to annotate the OTUs. The OTUs were classified and assigned using RDP classifier (version 2.2).^[^ ^56^ ^]^ The alpha‐diversity, beta‐diversity, and differential OTU abundance analysis were performed on the rarefied OTU table using QIIME (version 1.9.1).^[^ ^57^ ^]^ For indicator species analysis, the numbers of OUT sequences were used to obtain differential OUTs in R project edgeR package, indicspecies and labdsv packages in R project were used to calculate the indicator value of each species. The indicator values were finally tested with cross‐validation and presented as bubble plots.

### Untargeted Metabolome Analysis of Metabolites in Fecal Samples

Untargeted metabolome analysis of mouse fecal samples with different treatments was performed in Gene Denovo Biotechnology Co., Ltd. (Guangzhou, China). Briefly, ≈250 mg of mouse fecal samples from each replicate were used for metabolites extraction by adding of 300 µL of 80% methanol following previously published method.^[^ ^58^ ^]^ An internal standard for quality control (QC) was also included during sample preparation. Samples were successively homogenized using a homogenizer, sonicated for 10 min, incubated at −20 °C for 1 h, and centrifuged at 25 000 rpm for 15 min at 4 °C. Finally, the same volumes of samples and QC samples were transferred to autosampler vials for LC‐MS/MS analysis. To improve compound coverage, an ultra‐high‐performance liquid chromatography high‐resolution mass spectrometer (Thermo Scientific) was used to separate and detect positive and negative compound‐ion modes.

For data analysis, raw data files generated by UHPLC‐MS/MS were processed using Compound Discoverer 3.1 (Thermo Scientific) to perform peak alignment, peak picking, and quantitation for each metabolite. The parameters were set as follows: retention time tolerance, 0.2 min; actual mass tolerance, 5 ppm; signal intensity tolerance, 30%; signal/noise ratio, 3; and minimum intensity, 100 000. After that, peak intensities were normalized to total spectral intensity. The normalized data was used to predict molecular formula based on additive ions, molecular ion peaks and fragment ions. Finally, the identified peaks were matched with mzCloud (https://www.mzcloud.org/) and mzVaultand Mass Listdatabase. For visualization of differences between different groups of samples, the unsupervised dimensionality reduction method principal component analysis was applied in all samples using R package models (http://www.r‐project.org/). Metabolites with a VIP greater than 1, a *P* value of T test < 0.05, and log2 (foldchange) ≥ 1 were selected for differential analysis between samples. Enriched metabolites were mapped to KEGG for annotation and enrichment pathways analysis.

### Targeted Metabolome Analysis of Bile Acid Metabolites

Targeted metabolome analysis of mouse fecal samples with different treatments was performed in Shanghai Applied Protein Technology Co., Ltd. To extract metabolites from the samples, 800 µL of cold methanol/acetonitrile/water (2:2:1, v/v) extraction solvent was added to 100 mg sample, followed by homogenization, dissociation, and centrifugation. For absolute quantification of the metabolites, stock solutions of stable internal standards were added to the extraction solvent simultaneously. The LC–MS analysis was performed using an UHPLC (1290 Infinity LC, Agilent Technologies) coupled to a QTRAP MS (6500, SCIEX). MRM detection mode was used for mass spectrometry quantitative data acquisition of 350 targeted metabolites. Targeted metabolome analysis was performed in positive and negative switch mode. A polled quality control (QC) samples were set in the sample queue to evaluate the stability and repeatability of the system.

MultiQuant^[^ ^59^ ^]^ was used for quantitative data processing. The QCs were processed together with biological samples, and metabolites in QCs with coefficient of variation <30% were denoted as reproducible measures. For data processing, mean value of the metabolites was assigned in each group to the null value sample present in each group as the final level of that metabolite. After normalizing the metabolite levels in all samples, the R package model was used to perform principal component analysis on all samples to determine the variability of overall metabolite levels between groups. Metabolites with a *P* value < 0.05 and VIP value > 1 were considered as significantly differential metabolites between the FMT‐Amp group and FMT‐Con group. KEGG enrichment analysis was used to determine biological functions of final differential metabolites.

### m^6^A MeRIP‐Sequencing and RNA Sequencing

The m^6^A MeRIP‐seq libraries were built using the m^6^A‐seq2 protocol adapted from previous study.^[^ ^60^ ^]^ The m^6^A‐seq2 protocol employs multiplexed m^6^A‐immunoprecipitation of barcoded RNAs and pooled samples, and can greatly increase the throughput of samples. Before RNA barcoding and ligation, 1.8 µg N^−1^ (*N* indicates the number of samples for pooling) of poly‐A selected mRNA using Oligo d(T)25 Magnetic Beads (S1419S, NEB) was used for each sample at the beginning. RNA fragmentation at ≈150‐nt was performed using RNA fragmentation buffer (AM8740, Thermo Scientific). VAHTS RNA Clean Beads (N412, Vazyme Biotech) was used to purify RNA samples after fragmentation. For subsequent DNase and dephosphorylation treatment, each sample was incubated in T4 PNK (M0201, NEB), TURBO DNase (AM2238, Thermo Scientific) and FastAP (EF0651, Thermo Scientific) for 30 min in 37 °C in 5× FNK Buffer, followed by RNA cleanup using VAHTS RNA Clean Beads. 3′ RNA barcode adapter ligation was performed with 100 pmol of RNA ILL adapter (Table S8, Supporting Information) and 36U of T4 RNA ligase (NEB, M0204) for 1.5 h at room temperature for each sample. Following the 3′ ligation of barcoded RNA adapters, all samples were pooled for the multiplexed m^6^A‐IP and 10% of the sample pool was taken as the input‐RNA sample.

For multiplexed m^6^A‐IP, 40 µl of Protein G beads (10004D, Thermo Scientific) and 40 µl of Protein A beads (88 846, Thermo Scientific) were washed twice in 200 µl IPP buffer (10 mm Tris‐HCl, pH 7.5, 150 mm NaCl, 0.1% NP‐40 in RNase‐free water) and incubated with 4 µl rabbit anti‐m^6^A antibody (E1610S, NEB) for 6 h at 4 °C with rotation. RNA samples were denatured at 70 °C for 2 min and incubated with anti‐m^6^A‐Protein A/G beads for 2 h at 4 °C. After the incubation, RNA‐Protein A/G beads were washed twice with IPP buffer, low‐salt IPP buffer (50 mm NaCl, 0.1% NP‐40, 10 mm Tris‐HCl, pH7.5), high‐salt IPP buffer (500 mm NaCl, 0.1% NP‐40, 10 mm Tris‐HCl, pH7.5), respectively. RNA was eluted from the Protein A/G with 30 µl of RLT buffer (79 216, Qiagen), followed by RNA cleanup with VAHTS RNA Clean Beads. First strand cDNAs for m^6^A‐IP RNA or input‐RNA were syntheszed with rTd RT primer (Table S8, Supporting Information) and SuperScript III Reverse Transcriptase (18 080 051, Thermo Scientific). The cDNAs were purified with the MolPure PCR Purification Kit (19106ES50, Yeasen), followed by RNA hydrolysis in 1 m NaOH in 70 °C for 12 min and DNA cleanup using VAHTS DNA Clean Beads (N411, Vazyme Biotech). Illumina 5′adapter ligation was performed with 50 pmol 5iLL‐22 DNA adapter (Table S8, Supporting Information) with 45 U T4 RNA Ligase 1 (M0437M, NEB) for 6 h at 23 °C.

PCR enrichment was performed with KAPA HiFi PCR Kit (KK2601, KAPA Biosystems) with universal forward primer and reverse primer containing DNA barcode (Table S8, Supporting Information). Finally, amplified cDNA libraries were cleaned up and the library concentration was measured by Qubit 4 fluorometer. RNA‐seq and MeRIP‐seq of the prepared libraries were performed in Berry Genomics on the NovaSeq 6000 platform (Illumina, CA, USA) to obtain paired‐end reads of 150‐bp. Library quality was assessed on an Agilent Bioanalyzer 4200 TapeStation before loading onto the sequencer, ∼8G of raw reads was obtained for each demultiplex library.

### MeRIP‐seq Data Analysis

The raw data of MeRIP‐seq IP libraries were first processed with the same upstream pipeline employed for RNA‐seq. The mapping results of both MeRIP‐seq IP and input libraries were used for calling m^6^A peaks. Differential m^6^A peaks of the single‐factor comparisons between groups were analyzed using the R package exomePeak2 (version 1.6.1) (https://bioconductor.org/packages/release/bioc/html/exomePeak2.html) with a Poisson generalized linear model as the quantitative method. The “consistent_peak” option was used if applicable. To reduce false positives, the called m^6^A peaks from each biological replicate were considered as significant and retained for subsequent analyses according to the following thresholds: peak width ≤ 1500 bp, fold change ≥ 2, *P* < 0.05, and FDR < 0.05. Similarly, the called differential m^6^A peaks between groups were considered as significant under the following thresholds: peak width ≤ 1500 bp, fold change ≥ 1.5, *P* < 0.05, and FDR < 0.05. HOMER (version 4.11)^[^ ^61^ ^]^ was used for de novo motif searching around peak summit‐centered 300‐bp regions. The R packages ChIPseeker (version 1.18.0)^[^ ^62^ ^]^ and Guitar (version 2.10.0)^[^ ^63^ ^]^ were used for annotation and representation of the distribution characteristics of the significant/differential m^6^A peaks. PCA analyses of the Input data and IP data at the gene level (based on gene expression) were conducted. Integrative Genomics Viewer (IGV) software (version 2.12.2)^[^ ^64^ ^]^ was used to display read coverage tracks of target genes using the mapping results of both the IP and input libraries in bigWig format.

### RNA‐seq Data Analysis

Adaptors and raw reads containing low‐quality bases were first removed by applying Cutadapt (version 2.8) (https://doi.org/10.14806/ej.17.1.200) to all RNA‐seq raw data including the MeRIP‐seq input libraries. The resulting reads of at least 50 bp were mapped to the reference genome (mm39) released by UCSC database (Index of /goldenPath/mm39/bigZips). The featureCounts program^[^ ^65^ ^]^ in Subread package (version 2.0.2) at SourceForge was used to count the reads that mapped to genes. The genes that showed an average read count of less than 10 in any group or lacked read count in any replicate were primarily filtered. The R package DESeq2 (version 1.34.0)^[^ ^66^ ^]^ was used for the identification of differentially expressed genes (DEGs) by setting a false discovery rate‐corrected *P* value < 0.05 and fold change ≥ 2 as the thresholds for significance. R package DESeq2 was also used for read count normalization across samples by size factors and gene expression was measured based on normalized read counts.

### Label‐Free Quantitative Proteomics Analysis

Details of label‐free quantitative proteomic analysis have been reported in previous study.^[^ ^67^ ^]^ In general, the protocol can be divided into the following four parts: protein digestion and extraction, SDS‐PAGE, LC‐MS/MS analysis, protein identification, and quantification. First, SDT buffer (4% SDS, 100 mm Tris‐HCl, 1 mm DTT, pH7.6) was used for sample lysis and protein extraction, followed by protein separation using 12.5% SDS‐PAGE gel followed by Coomassie brilliant blue R‐250 staining. LC‐MS/MS analysis was performed on Q Exactive mass spectrometer with peptide recognition mode enabled. MaxQuant (version 1.5.3.17) was used to merge and search the raw MS/MS data of each sample for identification and quantitative analysis. Finally, the identified peptides were mapped to UniProtKB Swiss‐Prot database (Swissprot_Mus_musculus_17 063_20 210 106.fasta).

### LC‐MS/MS Quantification of m^6^A Modification Level

Purified mRNAs (≈200 ng for each sample) were digested in 30 µL reaction with 2 U nuclease P1 (N8630, Sigma), 250 mm NaCl, 25 mm ZnCl_2_ at 37 °C for 2 h, then 2 U FastAP (EF0651, Thermo Scientific) and 3.5 µL of 10× FastAP Buffer were added, followed by another 4 h of incubation at 37 °C. The digested nucleoside mixture was used for LC‐MS/MS quantification. Briefly, 5 µL of each digested sample was injected into a C18 reversed‐phase column coupled to an Agilent 6490 Triple Quad Mass Spectrometer (Agilent Technologies). In multiple reaction monitoring (MRM) positive electrospray ionization mode, the nucleosides were quantified by using retention time and nucleoside‐to‐base ion mass transitions (268.1–136.1 for A, 282.1–150.1 for m^6^A) with calibration curves from A (A9251, Sigma) and m^6^A standards (S3190, Selleck Chemicals). The ratio of m^6^A A^−1^ ratio was calculated based on the calibrated concentrations.

### Cell Culture and Bile Acids Treatment

Human cell lines (U251 cells, HepG2 cells, HCT116 cells) were grown in Dulbecco's Modified Eagle Medium (11965‐092, Gibco) supplemented with 10% fetal bovine serum (FBS500‐S, AUSGENEX) and 1% penicillin/streptomycin (15140‐122, Gibco) at 37 °C with 5% CO_2_. Lithocholic acid (Cat# A832268, Macklin), deoxycholic acid (Cat# D806701, Macklin), 7‐ketolithocholic acid (Cat# A184646, Aladdin), chenodeoxycholic acid (Cat# C104902, Macklin), and hyodeoxycholic acid (Cat# H106315, Macklin) were dissolved in DMSO. When cells were at the confluency of ≈70%, individual bile acid was added to cell culture medium at different concentrations (20, 100, and 500 µm). Control cells were only added with DMSO and all cells were collected for subsequent experiments after 24 h.

### Western Blotting Analysis

Fresh tissues or cells were homogenized using tissue homogenizer in 150 µl RIPA buffer (P0013B, Beyotime) and lysed on ice for 30 min. The suspension was centrifuged for 30 min at 13000× rpm, the supernatant was collected and protein concentrations of samples were measured by BCA assays (20201ES76, Yeasen). The samples were mixed with gel loading buffer and heated for 10 min at 105 °C. Protein samples were dissolved in 10% SDS‐PAGE and transferred to PVDF membrane (ISEQ00010, Millipore) for 1.5 h at 4 °C, followed by incubation with different primary antibodies. After incubation with secondary anti‐rabbit antibody (A0208, Beyotime) or anti‐mouse antibody (A0216, Beyotime), target proteins were detected using Clarity Western ECL Substrate (1 705 061, Bio‐Rad), and the intensities of protein bands were quantified using the ImageJ software and GraphPadPrism9.

### Immunofluorescence

Cells were grown in 12‐well tissue‐culture plates with slides and treated with bile acids. After 24 h, cells were fixed with 4% w/v formaldehyde (P0099, Beyotime), permeabilized with 0.5% v/v Triton X‐100 (DH351‐4, Dingguo Biotechnology Co., Ltd.) in phosphate‐buffered saline (C0221A, Beyotime) for 30 min. The slides were blocked for 30 min at room temperature in blocking buffer with 5% w/v goat serum (C0265, Beyotime). After blocking, the cells were incubated primary antibodies in blocking buffer overnight at 4 °C, and then incubated with goat anti‐rabbit IgG (H+L) Cross‐Adsorbed Secondary Antibody, Alexa Fluor 488 (A‐11008, Thermo Scientific) for 1 h at room temperature. Tissues were mounted in PBT buffer and stained with DAPI Staining Solution (P0131, Beyotime). A FLUOVIEW FV3000 confocal laser scanning microscope (Olympus, Japan) was used to capture immunofluorescence images.

### Antibodies

Primary antibodies used for Western blotting and Immunofluorescence are as follows: Rabbit anti‐METTL3 (Cat# 15073‐1‐AP, Proteintech), Rabbit anti‐METTL14 (Cat# HPA038002, Sigma), Mouse anti‐WTAP (Cat# 60188‐1‐Ig, Proteintech), Mouse anti‐FTO (Cat# ab92821, Abcam), Rabbit anti‐YTHDC1 (Cat# A7318, Abclonal), Rabbit anti‐METTL16 (Cat# A304‐192A, BETHYL), Rabbit anti‐Mat1a (Cat# 12395‐1‐AP, Proteintech), Rabbit anti‐Mat2a (Cat# 55309‐1‐AP, Proteintech), Mouse anti‐beta actin (Cat# ab6276, Abcam), Goat anti‐GAPDH (Cat# A00192, GenScript).

### Statistical Analysis

All data were expressed as the mean ± SD and representative data were shown. Statistical analyses used in this study include Wilcoxon test, Kruskal–Wallis, and two‐tailed Student's *t* test. The level of significance was set at *P* < 0.05; *P < 0.05; **P < 0.01; ***P < 0.001; ****P < 0.0001.

## Conflict of Interest

The authors declare no conflict of interest.

## Author Contributions

M.Y., X.Z., J.F., and W.C. contributed equally to this work. X.W., T.P., and H.W. conceptualized the study, designed experiments, and interpreted all the data. M.Y. and X.Z. performed all experiments with assistance from Y.L., N.Z., Y.L., J.Q., and Z.H. W.C. and H.W. helped with FMT experiments in GF mice. T.M.Y. helped with LC‐MS/MS quantification of m^6^A modification. M.Y. and J.F. performed all data analyses with help from Z.X., J.H., Y.J., B.L., R.P. and G.Z.L. X.W. and M.Y. wrote the manuscript with input from X.Z., S.H., G.L., C.J., A.M.E., E.B.C., and T.P. X.W. supervised and acquired funding for the study.

## Supporting information

Supporting Information

Supplemental Table 1

Supplemental Table 2

Supplemental Table 3

Supplemental Table 4

Supplemental Table 5

Supplemental Table 6

Supplemental Table 7

Supplemental Table 8

## Data Availability

The data that support the findings of this study are openly available in NCBI under accession numbers PRJNA902938, GSE218001, GSE216620. The mass spectrometry proteomics data have been deposited to the ProteomeXchange Consortium with the dataset identifier PXD038036.
