# Supplementary material for: Antibiotic‐Induced Gut Microbiota Dysbiosis Modulates Host Transcriptome and m6A Epitranscriptome via Bile Acid Metabolism
Source: Adv Sci (Weinh). 2024 May 7;11(28):2307981. doi: 10.1002/advs.202307981 (PMC11267274; doi:10.1002/advs.202307981)
Supplement: Supplementary file 1 — Supporting Information [file ADVS-11-2307981-s006.pdf]

ADVANCED SCIENCE
Open Access

Supporting Information

for *Adv. Sci.*, DOI 10.1002/advs.202307981

Antibiotic-Induced Gut Microbiota Dysbiosis Modulates Host Transcriptome and m^{6}A Epitranscriptome via Bile Acid Metabolism

Meng Yang, Xiaoqi Zheng, Jiajun Fan, Wei Cheng, Tong-Meng Yan, Yushan Lai, Nianping Zhang, Yi Lu, Jiali Qi, Zhengyi Huo, Zihe Xu, Jia Huang, Yuting Jiao, Biaodi Liu, Rui Pang, Xiang Zhong, Shi Huang, Guan-Zheng Luo, Gina Lee, Christian Jobin, A. Murat Eren, Eugene B Chang, Hong Wei, Tao Pan* and Xiaoyun Wang**

Supplementary Materials

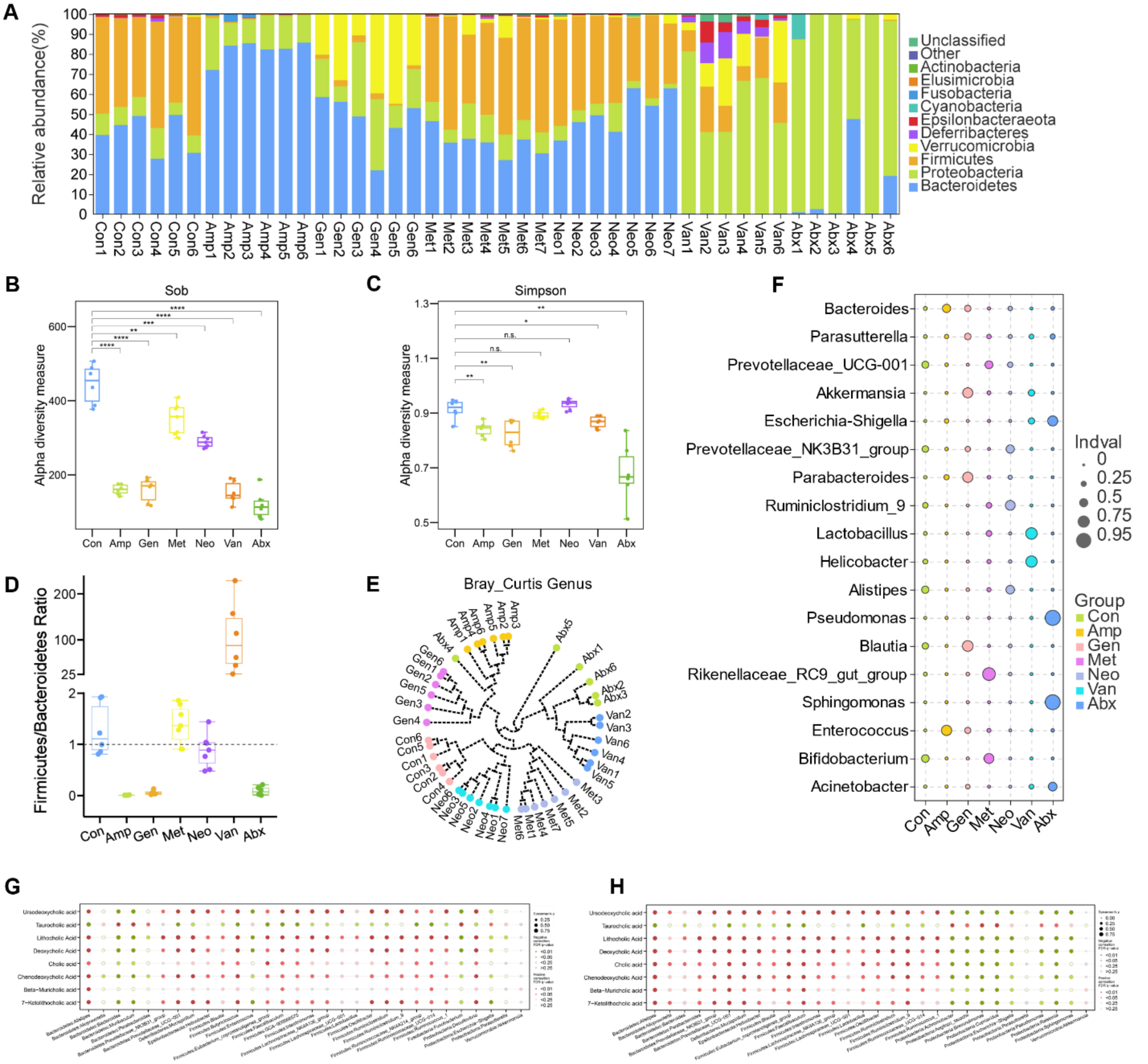

Figure S1. Microbial composition analysis of antibiotic-induced gut dysbiosis models (related to Figure 1). (**A**) Relative abundance of microbial compositions at phylum level in different antibiotic-treated groups (n = 6-7 each). (**B**) Sob index showing alpha-diversity of microbial compositions in different antibiotic-treated groups (n = 6-7 each). (**C**) Simpson index showing alpha-diversity of microbial compositions in different antibiotic-treated groups (n = 6-7 each). (**D**) *Firmicutes*/*Bacteroidetes* ratios in different antibiotic-treated groups (n = 6-7 each). (**E**) UPGMA analysis of microbial compositions at genus level in different antibiotic-treated groups (n = 6-7 each). (**F**) Indicator value analysis of microbial compositions at genus level in different antibiotic-treated groups (n = 6-7 each). (**G**) Correlation analysis of differential metabolites with the top 30 microbial compositions at genus level between Amp group and Con group (n = 5 each). (**H**) Correlation analysis of differential metabolites with the top 30 microbial compositions at genus level between Abx group and Con group (n = 5 each). Groups include control (Con), ampicillin (Amp), gentamicin (Gen), metronidazole (Met), neomycin (Neo), vancomycin (Van), antibiotic cocktail (Abx, mixture of the above five antibiotics). *P < 0.05, **P < 0.01, ***P < 0.001, ****P < 0.0001, Student's t test.

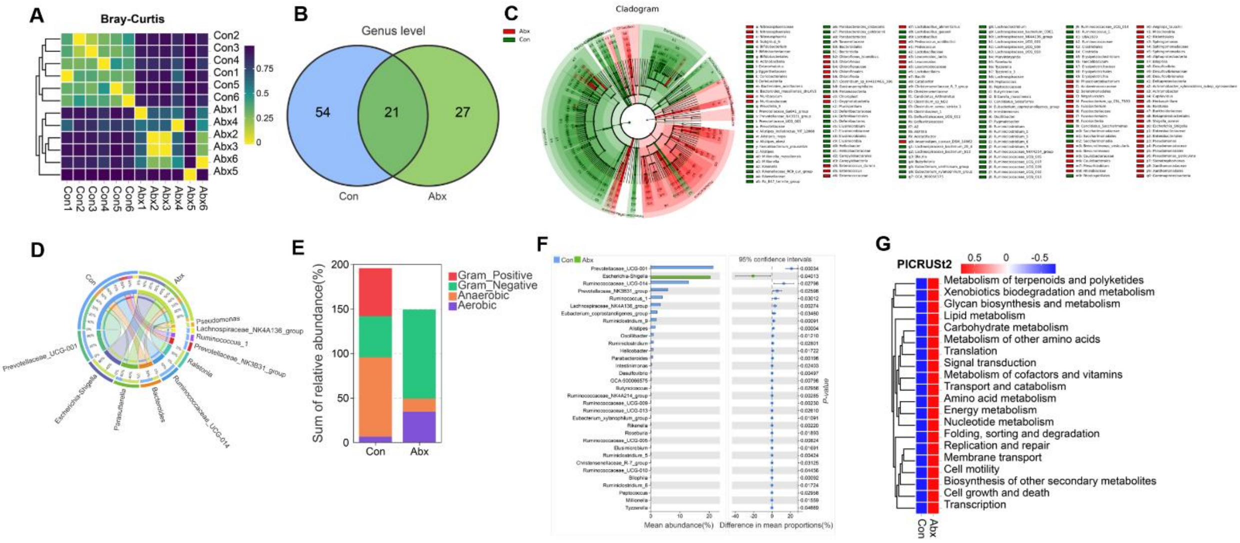

Figure S2. Microbial composition analysis for Abx group and Con group (related to Figure 1). (**A**) Pearson correlation heat map of 16S rRNA gene sequencing datasets showing microbial compositions in Abx group and Con group (*n* = 6 each). (**B**) Venn diagrams showing microbial compositions at genus level in Abx group and Con group. (**C**) LEfSe depicting taxonomic association of microbial compositions between Abx group and Con group. Each circle on a different taxonomic level represents a species under that taxonomic level and the diameter of the circle is proportional to the relative abundance. (**D**) Circos diagram depicting microbial compositions at phylum level in Abx group and Con group. (**E**) The phenotypic distribution of each group analyzed by BugBase. (**F**) Welch's t test showing differences in species abundance for Abx group and Con group. (**G**) Functional analysis on the gut microbiome for Abx group and Con group using PICRUSt2.

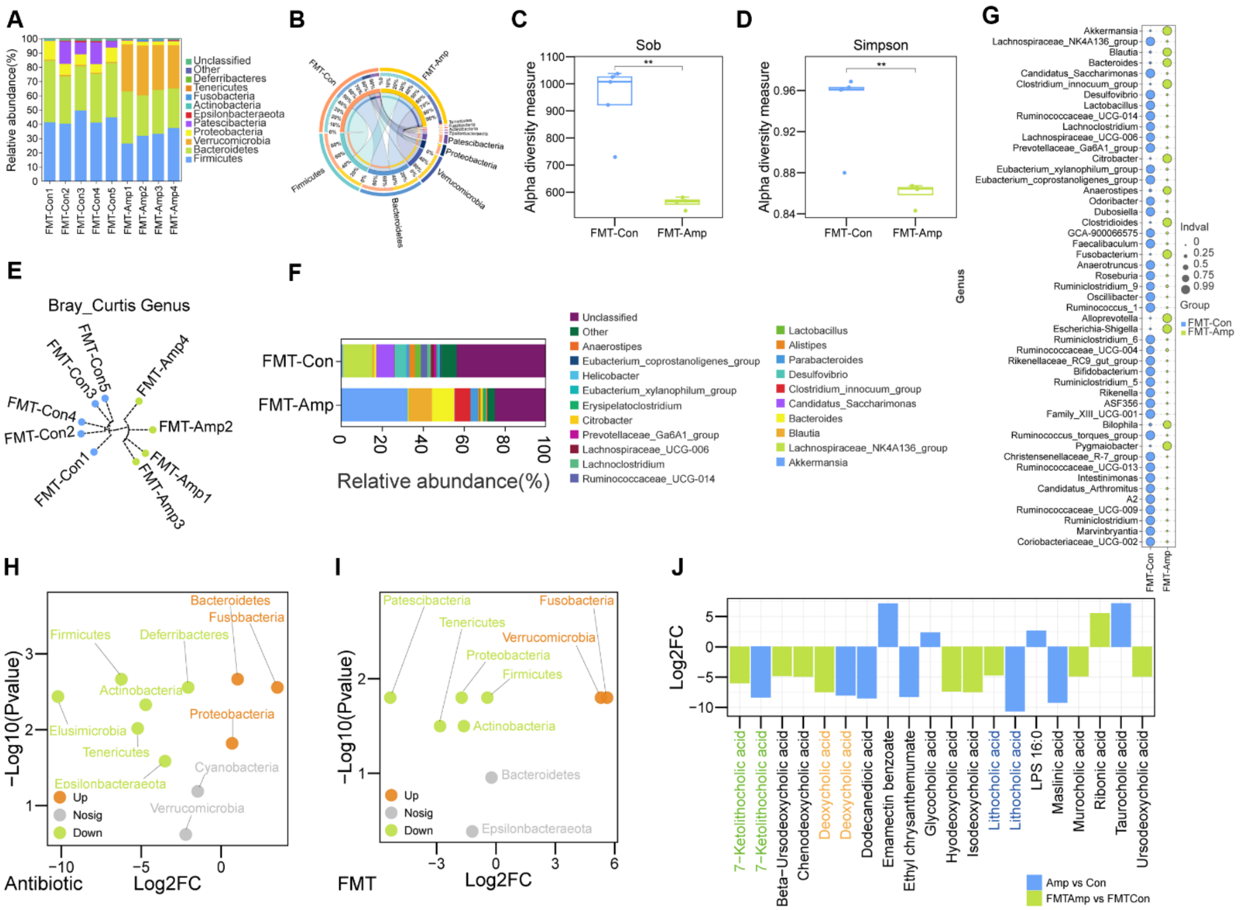

Figure S3. Microbial composition analysis after FMT experiments in germ-free mice (related to Figure 3). (**A**) Relative abundance of microbial compositions at phylum level in FMT-Amp group and FMT-Con group (*n* = 4-5 each). (**B**) Circos diagram depicting microbial compositions at phylum level in FMT-Amp group and FMT-Con group (*n* = 4-5 each). (**C**) Sob index showing alpha-diversity of microbial compositions in FMT-Amp group and FMT-Con group (*n* = 4-5 each). (**D**) Simpson index showing alpha-diversity of microbial compositions in FMT-Amp group and FMT-Con group (*n* = 4-5 each). (**E**) UPGMA analysis of microbial compositions at genus level in FMT-Amp group and FMT-Con group (*n* = 4-5 each). (**F**) Relative abundance of microbial compositions at genus level in FMT-Amp group and FMT-Con group (*n* = 4-5 each). (**G**) Indicator value analysis of microbial compositions at genus level in FMT-Amp group and FMT-Con group (*n* = 4-5 each). (**H**) Volcano plot showing up- and down-regulated microbiota at phylum level in Amp vs. Con comparison (*n* = 6-7 each). *P* value < 0.05, measured with the unpaired, two-tailed Student's t test. (**I**) Volcano plot showing up- and down-regulated microbiota at phylum level in FMT-Amp vs. FMT-Con comparison (*n* = 4-5 each). *P* value < 0.05, measured with the unpaired, two-tailed Student's t test. (**J**) Differential and common bile acids in Amp vs. Con comparison and FMT-Amp vs. FMT-Con comparison. Groups include control (FMT-Con) and ampicillin (FMT-Amp). **P* < 0.05, ***P* < 0.01, ****P* < 0.001, *****P* < 0.0001, Student's t test.

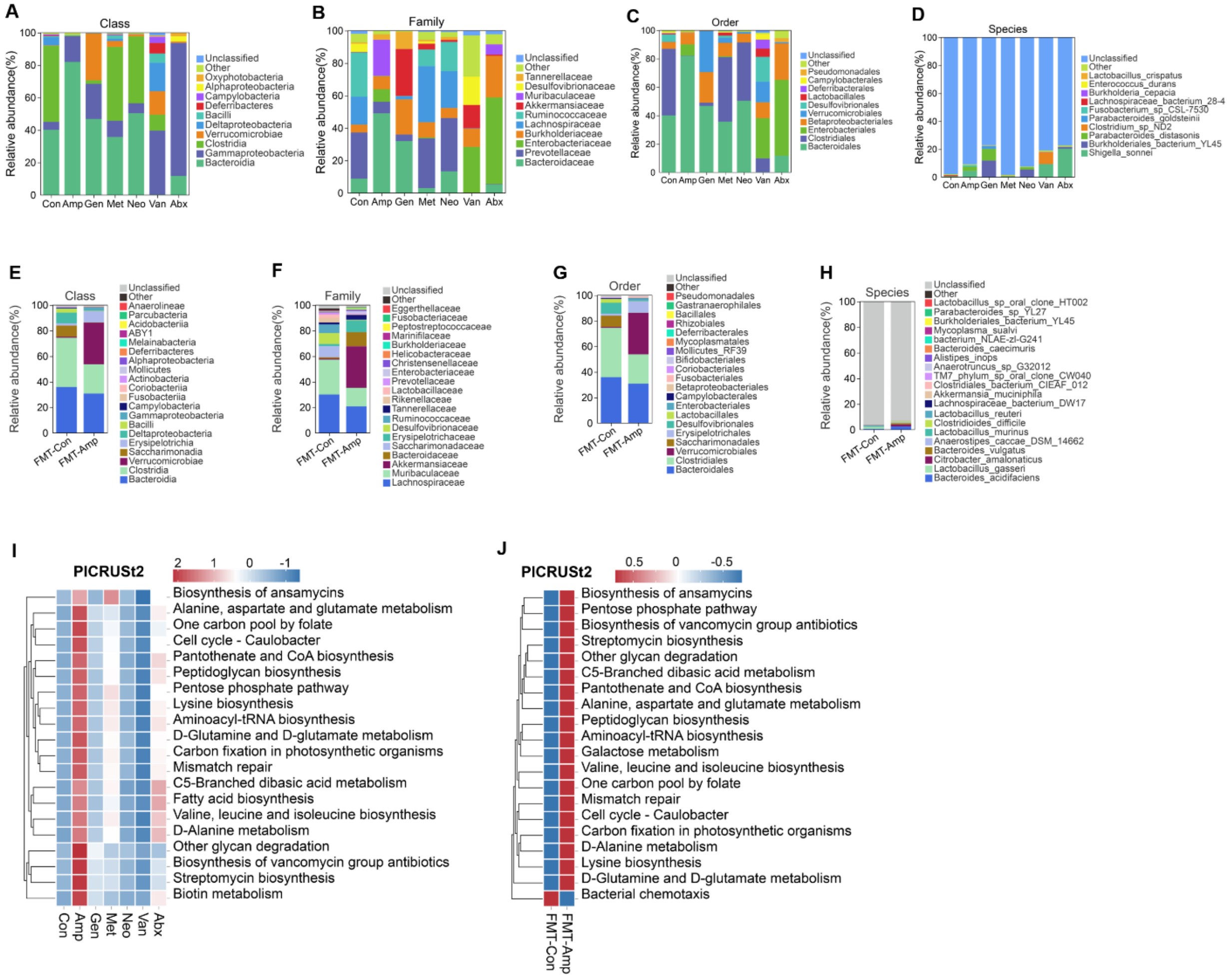

Figure S4. Microbial composition analysis at different levels (related to Figure 3). (**A-D**) Gut microbiota analysis of bacterial structure from SPF mice at class, family, order, species level, respectively (*n* = 6-7 each). Groups include control (Con), ampicillin (Amp), gentamicin (Gen), metronidazole (Met), neomycin (Neo), vancomycin (Van), antibiotic cocktail (Abx, mixture of the above five antibiotics). (**E-H**) Gut microbiota analysis of bacterial structure from GF mice at class, family, order, species level, respectively (*n* = 4-5 each). Groups include control (FMT-Con) and ampicillin (FMT-Amp). (**I**) Functional analysis on the gut microbiome for antibiotic-treated mice using PICRUSt2. (**J**) Functional analysis on the gut microbiome for FMT mice using PICRUSt2.

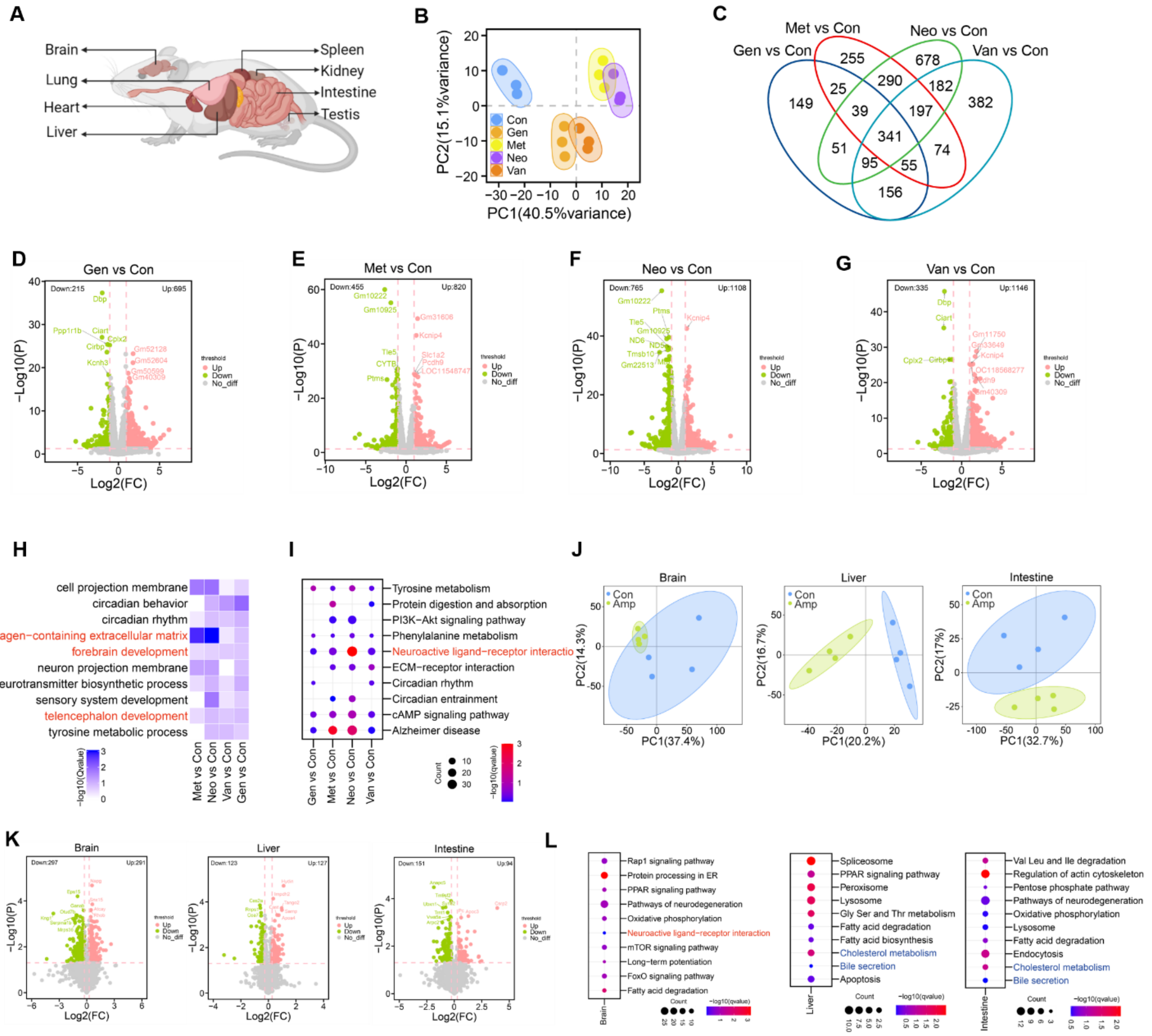

Figure S5. Transcriptome-wide impact of gut microbiota on brain gene expression (related to Figure 4). (**A**) Mouse model showing eight tissues from three groups (Con, Amp, Abx. *n* = 3 each) were collected for mRNA transcriptome sequencing. (**B**) PCA results of mouse brain mRNA transcriptome datasets in five groups (Con, Gen, Met, Neo, Van. *n* = 3 each). (**C**) Venn diagram showing the differences and overlaps of genes between groups (*n* = 3 each). (**D-G**) Volcano plot showing up- and down-regulated mouse brain genes in antibiotic treatment group compared to Con group (*n* = 3 each). Genes with expression level of fold change ≥ 2 and $P < 0.05$ are presented. (**H**) Gene Ontology analysis showing enriched pathways with differentially expressed genes in different groups compared to the control (*n* = 3 each). (**I**) KEGG analysis showing enriched pathways with differentially expressed genes in different groups compared to the control (*n* = 3 each). (**J**) PCA plot clustering mouse proteome datasets (brain, liver, intestine) between ampicillin treatment and the control (*n* = 4 each). (**K**) Differentially expressed proteins in proteome datasets (brain, liver, intestine) between ampicillin treatment and the control. Proteins with expression level of fold change ≥ 1.2 and $P < 0.05$ are presented. (**L**) KEGG analysis showing functional pathways of differentially expressed proteins in three tissues (brain, liver, intestine) by ampicillin treatment.

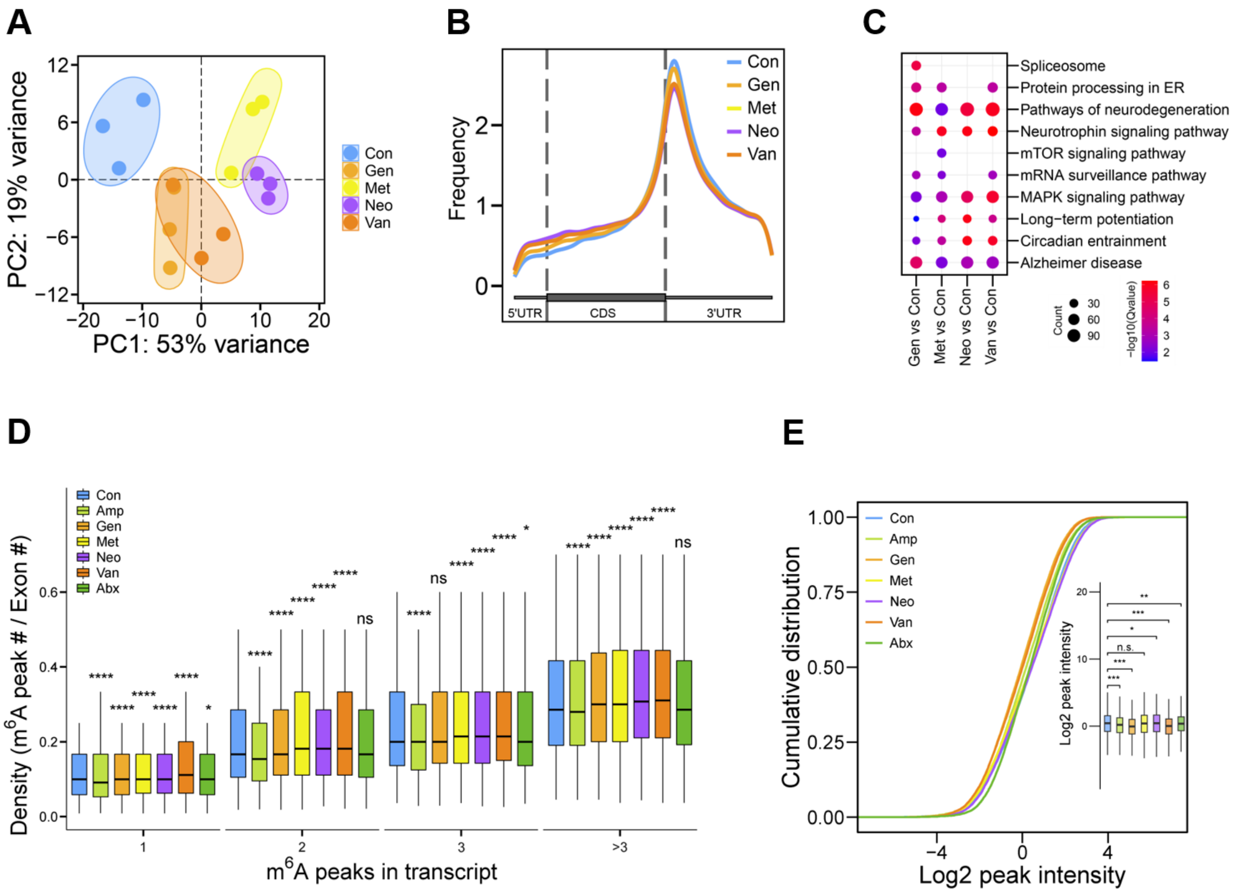

Figure S6. Gut dysbiosis rewires host brain mRNA m^{6}A epitranscriptome and gene expression (related to Figure 5). (**A**) PCA results of mouse brain mRNA m^{6}A epitranscriptome datasets in different groups (Con, Gen, Met, Neo, Van. *n* = 3 each). (**B**) The frequency of m^{6}A peaks distributed across the mRNA regions (5'UTR, CDS, 3'UTR) in different groups (*n* = 3 each). (**C**) KEGG analysis showing functional pathways of differentially abundant m^{6}A peak-containing genes between different antibiotic-treatment groups and the control (*n* = 3 each). (**D**) Statistical analysis of m^{6}A peak/exon density in m^{6}A peak-containing genes between control and each of six antibiotic-treatment groups (*n* = 3 each). (**E**) Cumulative distribution of m^{6}A peak intensity in mouse brain of seven treatment groups (*n* = 3 each). *$P < 0.05$, ** $P < 0.01$, ***$P < 0.001$, ****$P < 0.0001$, Mann-Whitney-Wilcoxon Test.

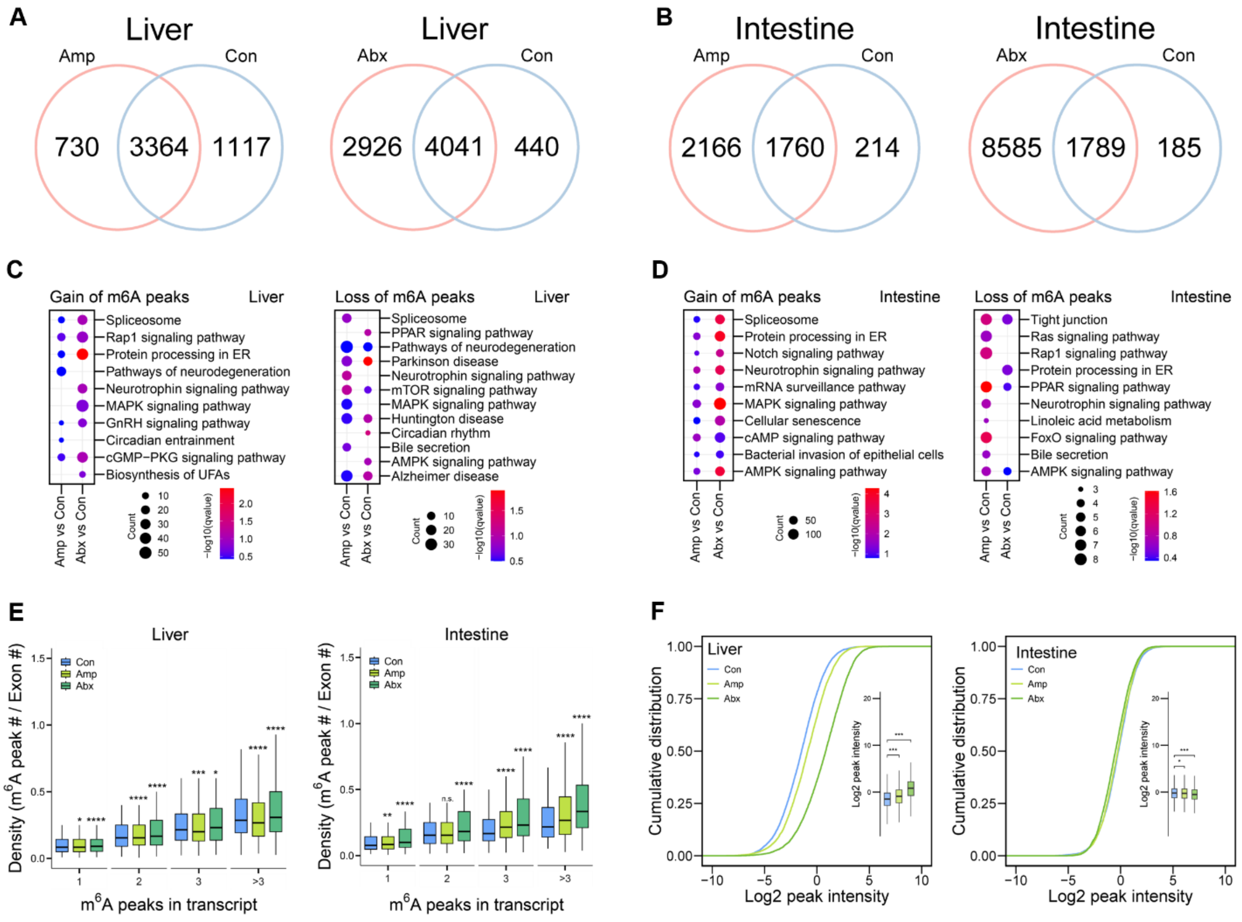

Figure S7. Gut dysbiosis rewires host liver and intestine mRNA m^{6}A epitranscriptome and gene expression (related to Figure 6). (**A**) The gain and loss peak numbers for the liver in three groups (Con, Amp, Abx. n = 3 each). (**B**) The gain and loss peak numbers for the intestine in three groups (Con, Amp, Abx. n = 3 each). (**C**) KEGG analysis showing pathways of liver genes gaining and losing m^{6}A peaks in different groups (n = 3 each). (**D**) KEGG analysis showing pathways of intestine genes gaining and losing m^{6}A peaks in different groups (n = 3 each). (**E**) Statistical analysis of m^{6}A peak/exon density in m^{6}A peak-containing genes (Con, Amp, Abx. n = 3 each). (**F**) Cumulative distribution of m^{6}A peak intensity in seven groups (Con, Amp, Abx. n = 3 each). *P < 0.05, ** P < 0.01, ***P < 0.001, ****P < 0.0001, Mann-Whitney-Wilcoxon Test.

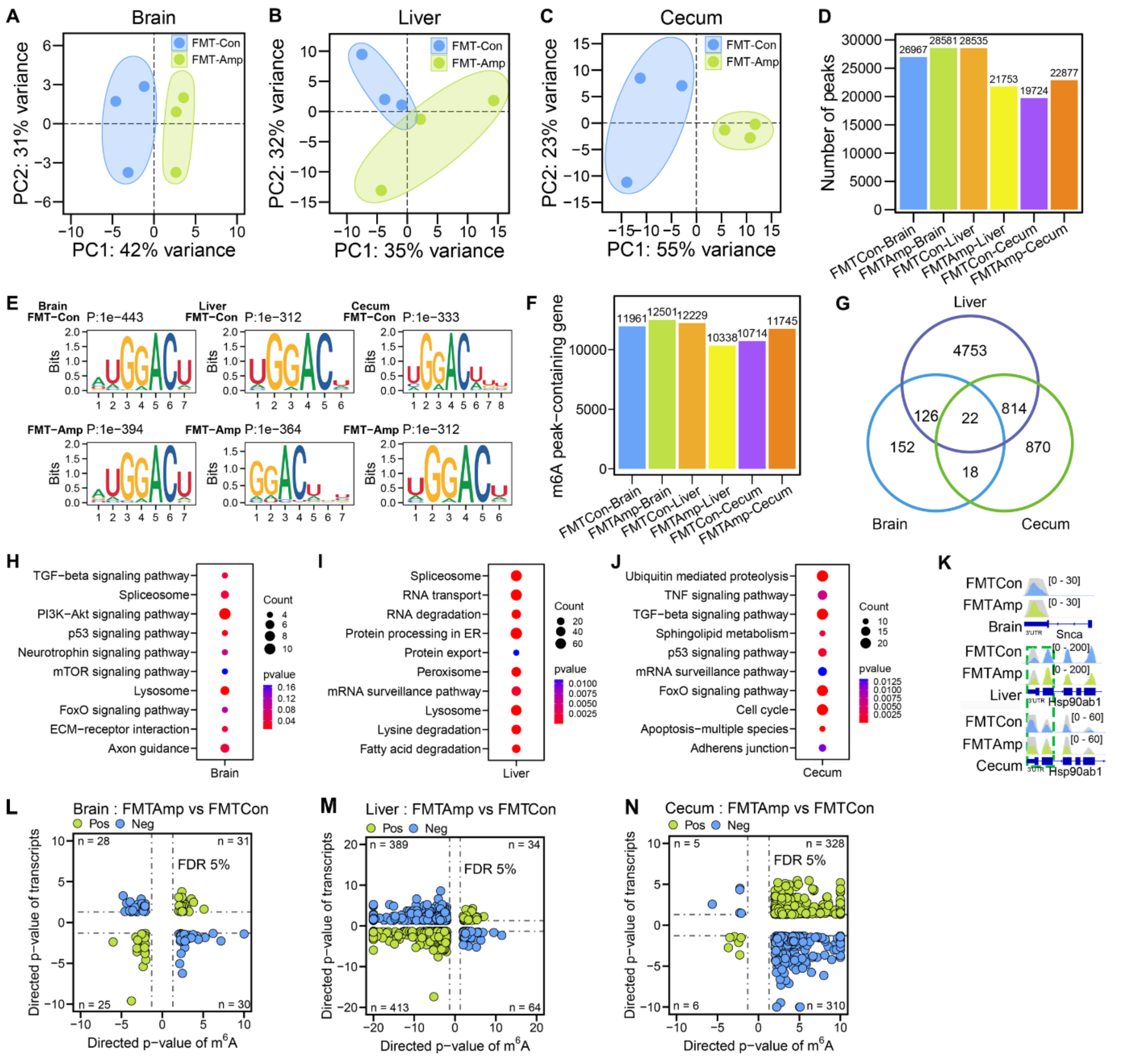

Figure S8. Gut dysbiosis reprograms host mRNA m^{6}A epitranscriptome in GF mice (related to Figure 6). (**A-C**) PCA plot clustering mRNA m^{6}A epitranscriptomes of three tissues (brain, liver, cecum) in FMT-Amp group and FMT-Con group (n = 3 each). (**D**) Numbers of peaks identified from brain, liver and cecum in two groups (n = 3 each). (**E**) Representative consensus motifs and corresponding P values of m^{6}A peaks identified from brain, liver and cecum in two groups (n = 3 each). (**F**) Numbers of m^{6}A peak-containing genes from brain, liver and cecum in two groups (n = 3 each). (**G**) Venn diagram showing the differences and overlaps of m^{6}A peak-containing genes between three tissues (n = 3 each). (**H-J**) KEGG analysis showing functional pathways of different m^{6}A peak-containing genes in three tissues (brain, liver, cecum. n = 3 each). (**K**) IGV depicting representative sequencing coverage of m^{6}A-IP (different colors) and Input (grey) showing differential m^{6}A peaks on transcripts *Snca* and *Hsp90ab1* in three tissues between two groups. (**L-N**) Correlation analysis of m^{6}A epitranscriptome with transcriptome in FMT-Amp compared to the control (brain, liver, cecum. n = 3 each).

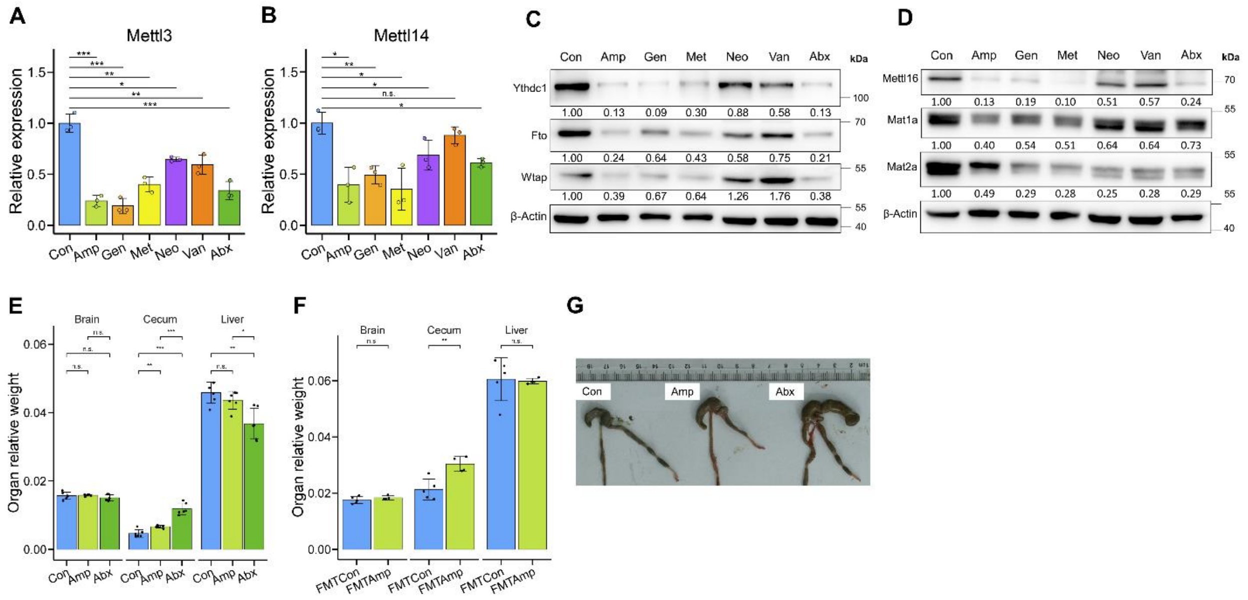

Figure S9. Gut dysbiosis regulates host m^{6}A writers in mouse tissues and associated phenotypes (related to Figure 7). (**A**) Quantitative analysis of relative expression of METTL3 in brain from different antibiotic treatments (Con, Amp, Gen, Met, Neo, Van, Abx. *n* = 3 each). (**B**) Quantitative analysis of relative expression of METTL14 in brain from different antibiotic treatments (*n* = 3 each). (**C**) Western blot analysis of mRNA m^{6}A machinery proteins (YTHDC1, FTO, WTAP) in brain from different antibiotic treatments, relative intensities of the proteins are indicated. (**D**) Western blot analysis of other methyl-donor related molecules (METTL16, MET1A, MET2A) in brain after antibiotic treatments, relative intensities of the proteins are indicated. (**E**) The organ relative weight of the tissues in mice after antibiotic treatments (*n* = 6). (**F**) The organ relative weight of the tissues in mice after FMT experiments (*n* = 4-5 each). (**G**) The images showing enlarged cecum tissue after antibiotic treatments. *P < 0.05, **P < 0.01, ***P < 0.001, ****P < 0.0001, Student's t test.

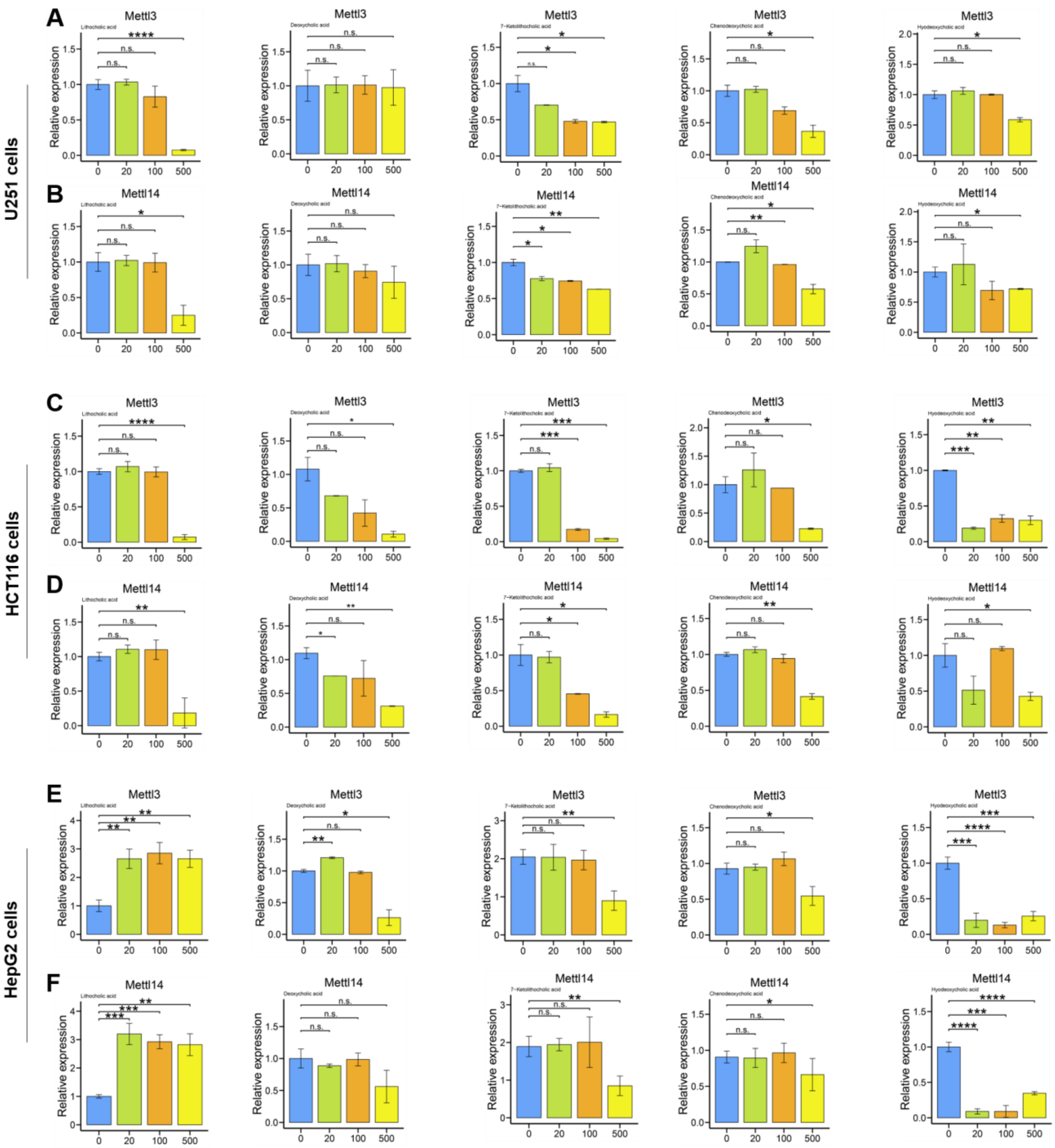

Figure S10. Bile acids treatments in mammalian cells alter the expression of m[6]A writers (related to Figure 8). (**A, B**) Quantitative analysis of relative expression of METTL3 and METTL14 in U251 cells treated with various concentrations of five different bile acids including lithocholic acid (n = 3), deoxycholic acid (n = 3), 7-ketolithocholic acid (n = 2), chenodeoxycholic acid (n = 2), and hyodeoxycholic acid (n = 2). (**C, D**) Quantitative analysis of relative expression of METTL3 and METTL14 in HCT116 cells treated with various concentrations of five different bile acids including lithocholic acid (n = 3), deoxycholic acid (n = 2), 7-ketolithocholic acid (n = 2), chenodeoxycholic acid (n = 2), and hyodeoxycholic acid (n = 2). (**E, F**) Quantitative analysis of relative expression of METTL3 and METTL14 in HepG2 cells treated with various concentrations of five different bile acids including lithocholic acid (n = 3), deoxycholic acid (n = 2), 7-ketolithocholic acid (n = 3), chenodeoxycholic acid (n = 2), and hyodeoxycholic acid (n = 3). *P < 0.05, **P < 0.01, ***P < 0.001, ****P < 0.0001, Student's t test.
